# Predictors of the Effectiveness of Psychedelics in Treating Depression—A Scoping Review

**DOI:** 10.3390/ijms27052202

**Published:** 2026-02-26

**Authors:** James Chmiel, Filip Rybakowski

**Affiliations:** 1 Faculty of Physical Culture and Health, Institute of Physical Culture Sciences, University of Szczecin, Al. Piastów 40B blok 6, 71-065 Szczecin, Poland; 2 Department and Clinic of Psychiatry, Poznan University of Medical Sciences, 61-701 Poznań, Poland

**Keywords:** psychedelics, depression, predictor, neuroplasticity

## Abstract

Psychedelic-assisted therapies (PATs) can produce rapid and sustained antidepressant effects, yet variability in response remains substantial. Identifying predictors and moderators is essential for optimising patient selection, preparation, and delivery. To map and synthesise the evidence on the predictors of antidepressant response to classic/serotonergic psychedelics administered with psychotherapeutic support in adults with depressive disorders, including treatment-resistant depression. Following PRISMA-ScR principles, we conducted a scoping review of major biomedical and psychology databases (PubMed (MEDLINE), Embase, PsycINFO, and Web of Science) and trial registries (searches September–October 2025), supplemented by reference-list screening. We included randomised trials, open-label studies, and naturalistic cohorts reporting associations between candidate predictors (baseline traits/clinical features, set/setting variables, acute in-session phenomenology, and biological measures) and validated depression outcomes. We charted study characteristics, analytic approaches (including moderation/mediation where available), and indicators of robustness (e.g., adjustment for overall intensity, preregistration, external validation). A total of 48 studies were included in the review. Across study designs, process-level features during the dosing session were the most consistent correlates of antidepressant improvement. Greater emotional breakthrough, mystical/unitive experiences, and ego dissolution-linked reappraisal/insight generally predicted larger and more durable symptom reductions, whereas anxiety-dominant or dysphoric states tended to attenuate benefit, often independent of overall subjective intensity. Set and setting—particularly a stronger therapeutic alliance and music experienced as resonant—predicted both the emergence of therapeutically salient acute experiences and downstream clinical gains. Baseline moderators showed smaller and mixed effects: PTSD comorbidity sometimes weakened trajectories; extensive prior psychedelic exposure was associated with smaller incremental gains; demographics were typically uninformative. Converging biological findings associated better outcomes with markers consistent with increased neural flexibility and plasticity (e.g., less segregated network dynamics; EEG indices), alongside peripheral changes implicating neurotrophic, inflammatory, and HPA axis pathways. Current evidence suggests that antidepressant response in PATs is driven less by static patient characteristics and more by what occurs during dosing and how the context shapes that experience. Optimising preparation, alliance, and music; facilitating emotional breakthrough and meaning making; and mitigating anxious dysregulation are actionable levers. Future trials should harmonise measures, pre-specify and validate moderators/mediators, intensively sample in-session experience and physiology, and report benefits and harms more consistently.

## 1. Introduction

Depression is one of the leading contributors to global disease burden, with a high lifetime prevalence and severe impacts on quality of life [1]. Standard antidepressant treatments (e.g., SSRIs) often have delayed efficacy and side effects—only about 35% of patients achieve full remission on initial therapy [2], and up to one-third develop treatment-resistant depression (TRD) after failing multiple adequate trials [3,4]. This substantial TRD population, alongside the modest effect sizes and high relapse/discontinuation rates of current antidepressants [5], underscores the need for new therapeutic strategies. In this context, classical psychedelics (such as psilocybin) have re-emerged as a potential intervention for refractory depression [6]. These substances showed promise in early trials of the 1960s before research was halted and are now experiencing a “renaissance” with modern clinical studies indicating significant antidepressant effects [7].

Accumulating evidence from recent trials and meta-analyses suggests that psychedelics—particularly psilocybin—can alleviate depressive symptoms, including in hard-to-treat cases. Multiple randomised controlled trials (RCTs) in patients with major depression have demonstrated that adjunct psilocybin therapy yields significantly greater symptom improvements than control conditions (placebo or conventional treatments) [8,9]. A 2023 meta-analysis pooling nine studies (n ≈ 596) found a large overall effect size favouring psilocybin over control (standardised mean difference ~−0.78, *p* < 0.001), with significantly higher response and remission rates under psilocybin-assisted therapy [10]. In a systematic review of five RCTs in depression (472 total patients), three trials (~60%) reported clear antidepressant benefits of psilocybin (versus active or inactive control), while two had mixed results [11]. Notably, even when psilocybin was compared head-to-head against an SSRI (escitalopram) in a double-blind trial, it was at least as effective in reducing depression severity and even outperformed the SSRI on several secondary outcome measures [12]. Furthermore, despite typically only one or two dosing sessions, psilocybin’s positive effects have shown surprising durability, with responders maintaining lower depression scores for 3–6 months or longer post-treatment in several studies [13,14].

**Figure 1:**
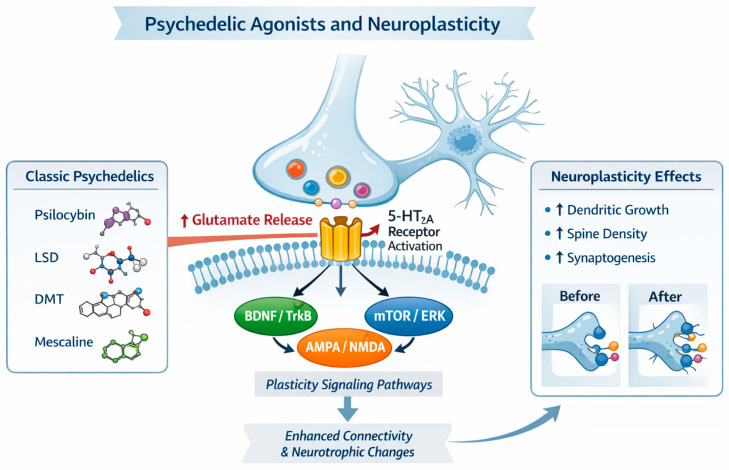
Mechanisms of action of psychedelics. ↑ increase.

Psychedelics exhibit a distinctive pharmacology and brain-wide impact that may underlie their antidepressant effects. Psilocybin’s active metabolite (psilocin) is a potent agonist at serotonin 5-HT_2_A receptors [15], which are densely expressed in cortical and limbic regions governing mood, cognition, and perception [16]. This 5-HT_2_A activation is considered a central trigger for downstream effects: it transiently “excites” frontal pyramidal neurons and initiates molecular cascades that enhance neuroplasticity [17]. For example, psychedelic 5-HT_2_A agonists induce glutamate release in prefrontal circuits [18] and activate AMPA-type glutamate receptors, in turn upregulating brain-derived neurotrophic factor (BDNF) and mTOR signalling pathways that promote synaptogenesis [19]. Psilocin can also directly bind to TrkB (the BDNF receptor), mimicking the pro-plasticity action of fast-acting antidepressants like ketamine [20]. These neurotrophic effects are supported by preclinical findings that psilocybin reverses stress-related synaptic deficits in limbic brain circuits implicated in depression [21]. Notably, while 5-HT_2_A stimulation is a key driver, it may not be the whole story: some evidence suggests that blocking 5-HT_2_A receptors does not fully abolish psilocybin’s antidepressant-like effects in animals, hinting that parallel pathways (e.g., 5-HT_1_A agonism or indirect dopamine/glutamate modulation) also contribute [22]. Beyond neurotransmitters, psychedelics produce other broad physiological changes consistent with antidepressant mechanisms. For instance, psilocybin transiently elevates plasma cortisol and other neuroendocrine markers [23,24], which might engage executive control circuits and emotional processing in beneficial ways. Concurrently, an anti-inflammatory signature has been observed: studies in healthy volunteers note reductions in pro-inflammatory cytokines (IL-6, TNF-α) and C-reactive protein after psilocybin administration [25]. This immunomodulatory effect could be relevant given the link between inflammation and depression [26]. The potential mechanisms of action of psychedelics are presented in Figure 1.

Although 5-HT_2_A activation is the canonical trigger for classic psychedelic effects, several other serotonergic (and non-serotonergic) targets are plausibly relevant to both acute phenomenology and downstream antidepressant processes. Receptorome and binding studies show that psilocin engages not only 5-HT_2_A but also 5-HT_1_A and 5-HT_2_C with comparable mid-nanomolar affinities, and classic psychedelics can additionally interact with 5-HT_2_B, 5-HT_7_, and other 5-HT_1_-family sites to varying degrees [27,28,29]. Functionally, 5-HT_1_A signalling is a plausible contributor to anxiolysis and affects regulation (potentially shaping the balance between “breakthrough” vs. dysphoric/anxious sessions), whereas 5-HT_2_C can modulate mesolimbic dopamine and stress responsivity—mechanisms that could influence acute emotional tone and learning-related plasticity [27,29]. Beyond serotonin, some psychedelics show meaningful activity at additional systems: for example, preclinical work supports the immunomodulatory actions of DMT/5-MeO-DMT via sigma-1 receptor signalling, and mescaline has been reported to show affinity at TAAR1, both of which could (at least theoretically) intersect with the inflammatory, arousal, and neuroplastic pathways implicated in depression [30].

Cardiac risk in psychedelic medicine is best separated into (i) acute hemodynamic effects and (ii) theoretical or substance-specific cardiotoxicity. Across controlled studies of classic psychedelics (e.g., psilocybin; also reported for LSD), the most consistent cardiovascular findings are transient, dose-related increases in heart rate and blood pressure that generally resolve as acute drug effects wane in screened participants under medical monitoring [31]. More serious events appear uncommon in modern trials, but case-based and safety reviews note that QTc prolongation can occur, particularly at higher exposures or in vulnerable individuals, supporting the standard practice of baseline cardiovascular screening, avoidance of QT-prolonging co-medications when possible, and ECG monitoring when clinically indicated [31]. A distinct concern relates to 5-HT_2_B receptor agonism, which is mechanistically linked to drug-induced valvular heart disease with chronic serotonergic stimulation (as seen historically with certain medications). Contemporary psychiatric protocols typically use intermittent dosing, which is expected to markedly reduce cumulative 5-HT_2_B exposure; however, authors have raised a plausible theoretical risk for high-frequency microdosing patterns, where repeated exposure could make valvulopathy a more relevant consideration [32,33,34]. Finally, it is important to distinguish “classic psychedelics” from certain other psychoactive compounds sometimes discussed alongside them: ibogaine has a comparatively strong and well-documented signal for clinically significant QT prolongation and ventricular arrhythmias (including torsades de pointes and cardiac arrest) in the case literature, warranting particular caution and a much higher monitoring threshold [35,36].

In clinical research, a predictor (also known as a prognostic or predictive factor) refers to a patient variable measured before or during treatment that is associated with subsequent outcomes [37]. In essence, predictors help forecast how a patient is likely to respond to a given therapy. There is a growing consensus that outcome research must move beyond asking “what works in general” to examining “what works for whom and under what circumstances” [38]. Predictors embody this shift by indicating which patients may benefit most from a treatment (or conversely, which may respond poorly), independent of any specific intervention effects. Identifying robust predictors allows clinicians to tailor interventions to individual patient needs [39]. Rather than a one-size-fits-all approach, treatment can be personalised based on a patient’s baseline characteristics or biomarkers. For instance, in psychiatry and psychology, research into therapy outcomes increasingly focuses on predictors to determine which patients will do better with a particular type of psychotherapy or medication [40,41]. These predictors can include clinical factors (e.g., symptom profiles, comorbidities), genetic markers, or even early treatment signals [42,43,44]. By stratifying patients in this way, clinicians can choose the most effective intervention for each person, improving overall efficacy, and avoiding unnecessary treatments. In effect, predictors answer the “who benefits?” question—a key principle of precision medicine and personalised care.

Predictors are also invaluable in clinical trial design and drug development. Knowing in advance which individuals are likely to experience the outcome of interest or to respond to the treatment can make trials more efficient and conclusive. Trials often employ stratified randomisation, ensuring that known prognostic factors (predictors of outcome) are balanced between treatment arms [45]. This improves internal validity by reducing outcome variability unrelated to the treatment. Moreover, investigators may use enrichment strategies that deliberately select participants based on predictor status to increase the trial’s power. Prognostic enrichment involves choosing high-risk patients who are more likely to have events (thereby increasing the event rate), whereas predictive enrichment selects patients who are biologically more likely to respond to the therapy (thereby amplifying treatment effects). By enriching the sample with likely responders, trials can demonstrate treatment benefits more clearly and with smaller sample sizes. In sum, leveraging predictors in trial design helps clarify “what works for whom” and accelerates the development of effective, targeted treatments.

Evaluating the validity of predictors is the central objective of this review article. As outlined above, predictors hold great promise for optimising treatments, but that promise hinges on evidence that the predictors are sound and useful. Indeed, there is no doubt that a systematic synthesis of treatment predictors and moderators is the first necessary step towards tailoring treatments for each person to maximise effectiveness. In the context of psychedelic therapy for depression, several candidate predictors (from patient demographics and genetics to psychometric or neuroimaging markers) have been suggested as factors that might influence treatment success. The critical question—and the focus of this review—is how robust these suggested predictors are. By rigorously reviewing the available studies, we aim to determine which predictors of psychedelic treatment effectiveness stand up to scrutiny in terms of reproducibility and clinical value. In other words, we will distinguish mere proposed predictors from those that have been validated as reliable indicators of who benefits from psychedelic therapy. This emphasis on validity is essential: only with well-validated predictors can clinicians confidently apply them to improve patient selection and outcomes in depression treatment. Establishing which predictors are truly evidence-based will help advance both the science of psychedelics in psychiatry and its translation into personalised clinical care.

## 2. Results

**Figure 2:**
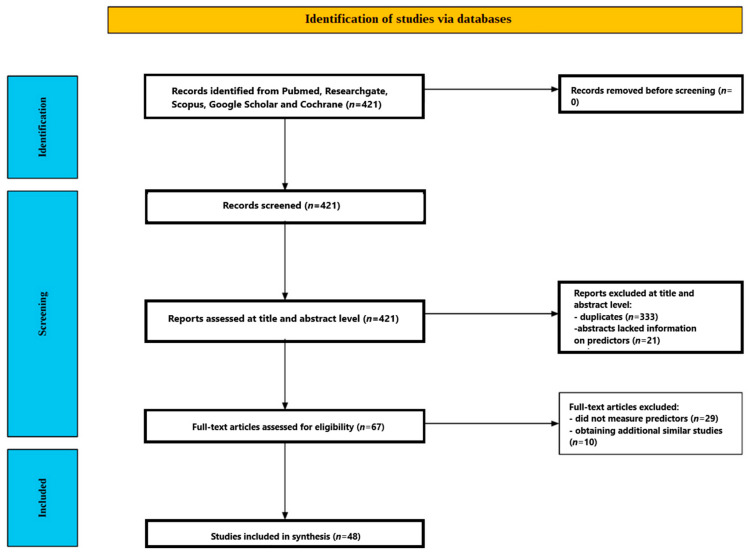
Flow chart depicting the different phases of the scoping review.

Figure 2 provides a summary of the screening process. Of the 421 studies initially identified through database research, 333 were excluded because they were duplicates. Titles and abstracts were reviewed for the remaining 88 studies. At this stage, 21 studies were excluded because the abstracts lacked information on the predictors and moderators of the effectiveness of psychedelic treatment for depression. Full-text analysis was performed for the remaining 67 studies. At this stage, 29 studies that did not measure predictors were excluded. A total of 38 studies were eligible. Searching for similar and cited articles and bibliographic searches of the identified studies yielded an additional 10 studies. Ultimately, 48 publications were included in the review [46,47,48,49,50,51,52,53,54,55,56,57,58,59,60,61,62,63,64,65,66,67,68,69,70,71,72,73,74,75,76,77,78,79,80,81,82,83,84,85,86,87,88,89,90,91,92,93]. The included studies are presented in Table 1.

### 2.1. Acute Psychedelic Experience as a Determinant of Antidepressant Response

Across trials, the quality of the acute psychedelic state—not merely its intensity—consistently tracked antidepressant benefit. In treatment-resistant depression (TRD), higher oceanic boundlessness (OBN; mystical/unitive experience) and lower dread of ego dissolution (DED; anxiety/impairment) recorded during dosing predicted larger QIDS-SR decreases at 1 day, 1 week, and 5 weeks, with large effects (partial η^2^ ≈ 0.25; *p* ≈ 0.002–0.003). OBN+DED jointly explained ~54% of the variance in 5-week symptom change; patients with a “complete” mystical-type experience (OBN > 0.6) showed consistently higher response rates across domains, whereas high DED was associated with poorer outcomes. Perceptual/auditory–visual changes did not predict improvement in this cohort, underscoring the specificity of emotional–existential content [33]. A dose-fixed psilocybin programme (25 mg; TRD and bipolar II) converged on this pattern after the first exposure: higher post-session MEQ-30 scores significantly predicted greater 2-week MADRS reductions (β = −0.387, *p* = 0.026) even after adjusting for baseline severity; this predictive link was absent after the second and third doses despite rising rates of “complete mystical experience” (29% after dose 1; 47% after dose 2; 60% after dose 3) [47].

Head-to-head randomisation against escitalopram (ET) showed that psilocybin therapy (PT) elicited stronger acute experiences on all key measures (mystical experience, ego dissolution, emotional breakthrough, emotional intensity). Mediation analyses identified mystical experience and ego dissolution as the only variables that significantly mediated PT’s advantage on depressive improvement; critically, these mediations held after controlling for general drug intensity, vivid visual imagery, and music impact—implicating experiential quality rather than mere strength. Within the PT arm, greater mystical experience, emotional breakthrough, and emotional engagement with music predicted larger week-6 depression decreases; the mediation effects were amplified in individuals with higher trait absorption/suggestibility [48]. In a larger phase IIb dose-finding trial (n = 233), although mean intensity rose with dose, there was substantial overlap across 1/10/25 mg. Across the full sample—and most clearly at 25 mg—OBN, visual restructuralisation, and Emotional Breakthrough Inventory (EBI) scores correlated strongly and negatively with 3-week MADRS change (r ≈ −0.51 to −0.64). By contrast, anxious ego dissolution and reduction in vigilance were only weakly related to improvement [49]. Earlier TRD work similarly found that “experience of unity,” “spiritual experience,” “blissful state,” and “insightfulness” during the high-dose session tracked larger 5-week depression decreases (e.g., r = −0.57 for insightfulness), leading the authors to treat these facets (“USB”) as a composite mediator of benefit [82].

Trials for cancer-related distress corroborate mystical-state mechanisms. In a double-blind crossover with high- vs. very low-dose psilocybin, MEQ-30 scores correlated strongly with 18 of 20 outcomes at five weeks (including GRID-HAMD and HAM-A) and significantly mediated psilocybin’s effect on seven prespecified endpoints (meaningfulness, spiritual significance, life satisfaction, and reductions in HADS anxiety, HADS depression, HADS total, and HAM-A), with confidence intervals excluding zero—even after adjusting for overall drug intensity [62]. A companion crossover showed parallel mediation by MEQ on mood outcomes (HADS, BDI, STAI) and durable effects up to 6.5 months [63]. In a randomised waiting list-controlled MDD trial, decreases in GRID-HAMD at four weeks correlated strongly with session ratings of personal meaning (r = −0.70, *p* < 0.01), psychological insight (r = −0.60, *p* < 0.01), and spiritual significance (r = −0.57, *p* < 0.01), and moderately with MEQ-30 (r = −0.41, *p* < 0.05); “complete mystical experience” per se was not significantly associated with depression change, and challenging experiences did not correlate with improvement [59].

Moderator analyses refine these associations. In severe TRD (single 25 mg), higher oceanic boundlessness predicted greater early improvement (week-1 MADRS r = −0.68, *p* = 0.016), but this association weakened by weeks 3 and 12; moreover, a significant interaction showed that OBN’s benefit was primarily present in participants without comorbid PTSD, while comorbid PTSD predicted weaker overall response (main effect and PTSD × time interaction) [61]. Two further datasets temper an exclusively “mystical” account. Under Switzerland’s medical-use programme (LSD or psilocybin), real-time session ratings indicated that relaxation was the strongest predictor of MADRS improvement; moment-to-moment acute effects explained ≈28% of outcome variance versus ≈4% for retrospective MEQ scores. Here, mystical-type experiences did not predict antidepressant change, and “complete” mystical experiences were rare [83]. Likewise, in U.S. veterans with severe TRD (73% with PTSD), robust MADRS reductions at 3 and 12 weeks (mean −23 points at week 3; d = 2.23, *p* < 0.001; response/remission 60%/53% at week 3; 47%/40% at week 12) were not mediated by total or subscale 5D-ASC scores. Notably, the week 3 association between total 5D-ASC and MADRS change *was* moderate in magnitude but non-significant (r = 0.41, *p* = 0.15) and was absent by week 12 (r = −0.13), consistent with limited power and/or a mechanism of antidepressant benefit not tightly coupled to acute phenomenology in this severe, highly comorbid+ veteran TRD sample [83].

In the study [53], participants reported markedly stronger altered states after psilocybin than after placebo (MEQ-30 mean = 70 ± 4.9 vs. 8.4 ± 4.5; *p* < 0.0001). Despite this, the intensity of mystical experience did not correlate with subsequent changes in depression scores following psilocybin dosing at any time point (1 day, 1 week, 2 weeks post-session). Interestingly, MEQ scores were negatively correlated with depression change after placebo—participants who reported more profound subjective experiences during the placebo session showed greater symptom reductions. This counterintuitive result emphasises that expectancy, psychological engagement, and contextual factors (rather than the pharmacological action of psilocybin itself) may substantially drive improvement.

Beyond mystical content, discrete experiential processes also carried predictive weight. In a large naturalistic study, when mystical-type experience (MEQ), challenging experience (CEQ), and emotional breakthrough (EBI) were modelled simultaneously, only EBI predicted greater 2-week depression reductions (β = −0.201, *p* = 0.039), implying that emotional processing/breakthrough is a key mediator when correlated constructs are held constant [64]. Complementing this, a randomised psilocybin-versus-SSRI trial found that greater ego dissolution and higher psychological insight were strongly associated with larger 6-week reductions in rumination (r = −0.44 and r = −0.69) and thought suppression (r = −0.41 and r = −0.56), cognitive shifts that themselves tracked symptom improvement (psilocybin arm: r = 0.48 and r = 0.49 with QIDS change) [76]. Finally, work in TRD highlighted the non-therapeutic role of anxiety-laden or unintegrated content: high DED predicted poorer outcomes [46], and in multiple datasets “challenging” experiences did not independently predict benefit once other experiential determinants (e.g., insight or EBI) were included [59,64]. Together, these findings indicate that the antidepressant signal is most strongly determined by acute states characterised by unitive/mystical qualities, ego dissolution-facilitated perspective shifts, and especially emotional breakthrough/insight, with anxiety-dominated or dysphoric experiences tending to attenuate, rather than enhance, clinical response.

Converging expectancy data echo this pattern: in a large psilocybin-user sample (n = 551), emotional breakthrough and ego dissolution—but not mystical experience when modelled simultaneously—uniquely predicted higher antidepressant expectancies (β = 0.32 and 0.21, both *p* < 0.001; mystical β = 0.07, ns), aligning perceived therapeutic value with the same qualitative features that track clinical improvement in trials [75].

### 2.2. Set and Setting: Therapeutic Alliance and Music

A strong therapeutic relationship and a well-matched musical environment emerged as precise, process-level determinants of antidepressant response. In the randomised comparison of psilocybin therapy (PT) versus escitalopram, analyses focused on the PT arm (n = 30) showed that a stronger therapeutic alliance (STAR-P) before dosing predicted greater emotional breakthrough (EBI) and mystical-type experience (MEQ) during sessions and lower depression six weeks later; path models indicated that both EBI and MEQ mediated the alliance → outcome pathway, with emotional breakthrough explaining slightly more variance (R^2^ = 0.42) than mystical experience (R^2^ = 0.32). Session-by-session effects were differentiated: in session 1, EBI strongly mediated the alliance–improvement link and also predicted a stronger alliance prior to session 2; in session 2, mystical-type experience (rather than EBI) predicted further depressive symptom reductions, and a weaker pre-session alliance forecast higher endpoint depression [60].

Independent evidence from a randomised, waitlist-controlled MDD trial reinforced these effects and extended them longitudinally. The alliance increased from the final preparation visit to 1-week post-psilocybin (*p* = 0.027, Cohen’s d = 0.43), driven by the “task” component (*p* < 0.001, d = 0.65). A stronger pre-session alliance correlated with larger GRID-HAMD reductions at 4 weeks (r = −0.65, *p* = 0.002), 6 months (r = −0.47, *p* = 0.036), and 12 months (r = −0.54, *p* = 0.014). Post-session alliance was an even stronger predictor: 4 weeks r = −0.85 (*p* < 0.001), 6 months r = −0.77 (*p* < 0.001), and 12 months r = −0.61 (*p* = 0.001). Mechanistically, higher pre-session alliance correlated with more intense acute experiences (MEQ r = 0.49, *p* = 0.027; psychological insight r = 0.52, *p* = 0.040), and psychological insight tracked depression improvement most strongly across follow-ups (e.g., r = −0.75 at 4 weeks, *p* < 0.001; r = −0.70 at 12 months, *p* < 0.001) [69].

Music—the other major element of “setting”—showed a parallel, experience-shaping role. In the PT vs. SSRI trial, emotional engagement with music during dosing predicted larger week-6 depression decreases; crucially, mystical experience and ego dissolution still mediated PT’s advantage after controlling for general drug intensity, vivid visual imagery, and music impact, indicating that it was the quality of the psychedelic state (not mere intensity or soundtrack) that explained benefit [48]. In a targeted study of music with TRD patients, independent ratings of liking, resonance (fit with inner state), and openness one week after treatment showed that the quality of the music experience predicted both the mystical experience and insightfulness and the reduction in depressive symptoms at 1 week; by contrast, self-rated drug intensity correlated only with perceptual changes and impaired cognition, not with therapeutic outcomes [66].

Finally, the emotional tone of integration conversations captured the alliance’s downstream effect on change. In a phase IIb secondary analysis using transcripts from integration sessions held 1 day after dosing (n = 101; 90 with week-12 data), higher EBI scores, more positive valence and greater arousal in both participant and therapist speech, and higher dose were each independently associated with better outcomes. Logistic models predicted week-3 responders with pseudo-R^2^ = 0.51 (leave-one-out accuracy 85%, AUC 0.88) and sustained responders to week 12 with pseudo-R^2^ = 0.44 (accuracy 88%, AUC 0.85) [70].

### 2.3. Expectancy, Suggestibility, and Absorption

Across head-to-head and secondary analyses, expectancy effects were robust for SSRI comparators yet largely absent for psilocybin, whereas suggestibility (but not absorption) reliably moderated psilocybin benefit. In the imperial randomised trial re-analyses, participants entered with markedly higher improvement expectancies for psilocybin than for escitalopram (mean ≈ 54% vs. 28% expected improvement on 0–100 VAS) across arms. Within the escitalopram arm, higher pre-treatment expectancy predicted greater symptom improvement over time on multiple scales, including the HAM-D, BDI, MADRS, and STAI-T (e.g., +1 SD in escitalopram expectancy—~22 VAS points—→ 3.9-point larger HAM-D reduction). In contrast, psilocybin expectancy did not significantly predict outcomes on any of these measures. When expectancy was covaried, the nominal between-treatment advantage for psilocybin disappeared, consistent with a nocebo effect penalising escitalopram rather than a placebo boost for psilocybin; by comparison, adjusting for suggestibility (instead of expectancy) left psilocybin’s advantage intact on nearly all scales. Baseline suggestibility (Short Suggestibility Scale) showed a treatment-specific pattern: in the psilocybin arm only, each +1 SD (~10 points) corresponded to an additional ~3.5-point HAM-D reduction, with parallel benefits on BDI, MADRS, QIDS-SR-16, STAI-T, and WEMWBS; absorption (MODTAS) did not predict outcomes in either arm [65].

A preregistered secondary analysis focusing on expectancy replicated and extended these findings. There was a significant treatment × escitalopram expectancy interaction for the primary HDRS-17 endpoint at 6 weeks (β = −0.106, SE = 0.033, *p* = 0.002): when escitalopram expectancy was low, psilocybin yielded much lower HDRS-17 scores; as escitalopram expectancy approached 80–100%, arm differences converged. The same interaction held for MADRS (β = −0.153, SE = 0.057, *p* = 0.009) and BDI (β = −0.154, SE = 0.070, *p* = 0.03), but not QIDS-SR-16 (β = −0.064, SE = 0.037, *p* = 0.09). Within groups, escitalopram expectancy correlated with final HDRS-17 (r = −0.49, *p* = 0.008), whereas outcomes among psilocybin recipients were not related to psilocybin expectancy (r = 0.30, *p* = 0.14). Median (IQR) expectancies were 23% (11–50%) for escitalopram and 60% (40–71%) for psilocybin; the two expectancy ratings were only modestly related (r = 0.25, *p* = 0.06) [71].

Personality change analyses from the same trial programme showed an expectancy-specific influence in the SSRI arm: higher escitalopram expectancy predicted greater decreases in neuroticism (β = −0.01 per expectancy unit, *p* = 0.002) and greater increases in conscientiousness (β = 0.01, *p* = 0.004). No expectancy effects on personality were detected for psilocybin; between-arm differences in personality change were small, with a trend toward higher absorption after psilocybin (B = 0.23, *p* = 0.037, below the conservative threshold for non-hypothesised outcomes) [72].

Complementing trial-based expectancy analyses, a large cross-sectional study of psilocybin users (n = 551) mapped what people expect to improve with psilocybin-assisted therapy and which experiences those expectations are anchored to. Antidepressant expectancies correlated more strongly with ego dissolution (r = 0.50) and emotional breakthrough (r = 0.52) than with mystical-type experience (r = 0.38), and far more than with demographics, current symptoms, or lifetime hallucinogen involvement (|r| ≤ 0.21). In a joint model, only emotional breakthrough and ego dissolution uniquely predicted higher antidepressant expectancies (β = 0.32 and 0.21, *p* < 0.001), with a small contribution from current depressive severity (β = 0.14, *p* < 0.001); mystical experience did not add unique variance. Expectancies were symptom-specific, favouring affective domains (hopefulness, mood, fear) over vegetative/behavioural ones (sleep, concentration, activation): paired-index contrast t(550) = 15.51, d = 0.66. The rank order of expected symptom improvements closely paralleled ayahuasca findings (Spearman ρ = 0.73), indicating a shared expectancy template across serotonergic psychedelics [75].

Finally, the moderation of acute experience mediation by trait variables was observed within the psilocybin condition: mystical experience and ego dissolution mediated psilocybin’s superiority over escitalopram for depressive improvement, and these indirect effects were strongest in participants with higher trait absorption and suggestibility—even after adjusting for general drug intensity, vivid imagery, and music impact—implicating a synergy between set (traits that enable immersive engagement) and state (qualitative features of the session) in driving therapeutic gain [48].

### 2.4. Dose, Dosing Schedule, and Prior Use

Across trials, the dose shaped outcomes primarily by modulating qualitative features of the acute experience rather than by sheer intensity, and the first session carried the strongest predictive signal. In the phase IIb dose-finding trial (n = 233), mean psychedelic intensity increased from 1 → 10 → 25 mg COMP360 (COMPASS Pathways’ proprietary, synthesised (lab-made), purified formulation of psilocybin), but overlapped widely between groups; nonetheless, specific experiential dimensions—Oceanic boundlessness, visual restructuralisation, and emotional breakthrough (EBI)—showed the tightest associations with improvement at 3 weeks, correlating negatively with MADRS change across the full sample and most clearly at 25 mg (r ≈ −0.51 to −0.64), whereas anxious ego dissolution and reduction in vigilance were only weakly related [49]. In a fixed-dose (25 mg) programme with up to three psilocybin sessions, the first dose’s mystical intensity (MEQ-30) significantly predicted the 2-week MADRS reduction (β = −0.387, *p* = 0.026; baseline-adjusted), but this predictive link did not recur after the second or third doses despite rising rates of “complete mystical experience” (29% → 47% → 60% across doses); persistence of complete mystical states across sessions was observed, but small multi-dose subsamples (dose-2 n = 17; dose-3 n = 5) limited power [47]. Naturalistic data converged: higher self-reported dose predicted larger 2-week QIDS decreases (β = 0.196, *p* = 0.029), while prior psychedelic experience predicted smaller improvements (β = 0.213, *p* = 0.033), and most symptomatic gain occurred rapidly with little additional change between weeks 2 and 4 [64]. A single-session clinical context underscores these dynamics: in U.S. veterans with severe TRD given one 25 mg dose, MADRS fell by 23 points at week 3 (d = 2.23, *p* < 0.001), with 60% response and 53% remission (week 12: 47%/40%), yet neither total nor subscale 5D-ASC scores significantly mediated these changes [83]. Collectively, these findings suggest that higher doses tend to yield greater antidepressant benefit when they foster unitive or transformational experiences; however, interindividual variability is substantial, the first session’s experiential quality most strongly predicts short-term response, and extensive prior psychedelic exposure may attenuate incremental gains.

### 2.5. Comorbidity and Baseline Characteristics

Comorbidity—especially post-traumatic stress disorder (PTSD)—and several baseline psychological features systematically moderated antidepressant outcomes. In a severe TRD cohort treated with a single 25 mg psilocybin dose, comorbid PTSD (5/12 participants) was associated with a weaker antidepressant trajectory on the MADRS: linear mixed models showed a main effect of PTSD and a PTSD × time interaction, with higher MADRS scores at weeks 6 and 12 in the PTSD subgroup; notably, all seven participants without PTSD met response or remission, whereas only a subset with PTSD did so. Within-session oceanic boundlessness (OB) predicted early improvement (week-1 MADRS r = −0.68, *p* = 0.016), but this association diminished by weeks 3–12 and was contingent on PTSD status (benefit of OB evident primarily when PTSD was absent) [61]. By contrast, in a U.S. veteran sample with severe TRD—73% meeting PTSD criteria—one 25 mg psilocybin session produced large and durable improvements (mean MADRS −23 at week 3; d = 2.23, *p* < 0.001; 60% response/53% remission at week 3; 47%/40% at week 12), and outcomes were not detectably influenced by age, sex, race, or PTSD; neither total nor subscale 5D-ASC scores mediated change [83]. In the head-to-head psilocybin-versus-escitalopram trial, demographic variables (age, sex) likewise showed no predictive value for symptom change, whereas acute experiential quality did [48].

Baseline linguistic and personality markers also carried prognostic signals. In a pre-treatment autobiographical speech task, patients used fewer positive words than healthy controls (AVG *p* = 0.038 vs. 0.053; *p* = 0.0011); a Gaussian Naive Bayes model separated patients from controls with ~83% accuracy and, critically, predicted responders vs. non-responders at ~85% accuracy (precision = 0.75). Responders tended to use fewer emotional—particularly positive—words at baseline, indicating greater potential for affective improvement [54]. In a TRD personality study, psilocybin produced decreased neuroticism and increased extraversion/openness at 3 months; insightfulness during dosing predicted both neuroticism reduction and extraversion increase, while spiritual experience correlated with extraversion gains. Exploratory analyses indicated that higher baseline neuroticism tended to track less depressive improvement [55]. Complementing this, in the psilocybin-versus-SSRI RCT, trait suggestibility (baseline Short Suggestibility Scale) predicted greater psilocybin benefit across all symptom scales (≈3.5-point additional HAM-D reduction per +1 SD in suggestibility), whereas absorption did not predict outcomes in either arm [65].

Motivational and exposure history at baseline further shaped effects—most clearly in large naturalistic datasets. When controlling for age, gender, education, and baseline severity, a medicinal motive (self-healing/therapy) predicted larger QIDS reductions (β = 0.316, *p* = 0.002), higher self-reported dose predicted additional improvement (β = 0.196, *p* = 0.029), and previous psychedelic experience predicted smaller gains (β = 0.213, *p* = 0.033). Stratified by initial severity, participants with moderate depression improved from 12.6 → ~7.0, and those with severe depression from ~18.5 → 6.3 at 2–4 weeks (effect sizes in moderate-to-severe: d = 2.4 at 2 weeks; d = 2.1 at 4 weeks) with only a non-significant relapse tendency in the severe subgroup between weeks 2 and 4 [64]. In an open-label psilocybin programme, history of abuse predicted stronger reductions in PTSD and neuroticism; ongoing professional support outside the programme enhanced long-term anxiety reduction; lower alcohol use was associated with greater improvements in PTSD, neuroticism, openness, and conscientiousness; younger age predicted larger anxiety decreases. Higher neuroticism may contribute to long-term anxiety persistence via increased stress reactivity and negative repetitive thinking (worry/rumination) and through related constructs such as intolerance of uncertainty, which together can maintain anxious arousal and avoidance over time. Session-level surrender and emotional engagement robustly predicted improvements across depression, anxiety, PTSD, and personality; within the MEQ-30, transcendence of time/space/self most strongly moderated gains in depression/anxiety and in openness/conscientiousness [82]. Relatedly, an expectancy study found minimal links between demographics or lifetime psychedelic exposure and antidepressant expectancies, with only current depressive symptoms showing a small positive association (β = 0.14, *p* < 0.001)—suggesting that baseline clinical burden (but not sociodemographics or prior use) modestly inflates the expected benefit [75]. Taken together, these findings indicate that (i) PTSD comorbidity can attenuate response and blunt the benefit of unitive states in some TRD samples, though not universally; (ii) pre-treatment speech and personality/trait suggestibility provide actionable prognostic information; and (iii) initial motive, substance use context, and ongoing support meaningfully shape the magnitude and durability of antidepressant benefit.

### 2.6. Biological Mediators and Moderators

Across ayahuasca RCTs and secondary analyses, antidepressant improvement co-occurred with specific neuroendocrine and inflammatory shifts—it most consistently increased brain-derived neurotrophic factor (BDNF), reduced C-reactive protein (CRP), and normalisation of hypothalamic–pituitary–adrenal (HPA) axis dynamics—while interleukin-6 (IL-6) showed null effects.

First is neurotrophins (BDNF) and cortisol context. In a double-blind randomised placebo-controlled trial, baseline serum BDNF did not predict depression severity; instead, serum cortisol status predicted BDNF: patients with hypocortisolaemia had lower BDNF than eucortisolaemic patients, and among eucortisolaemic volunteers cortisol correlated negatively with BDNF, implying an inverted-U cortisol–BDNF relation [57]. Forty-eight hours after dosing, ayahuasca (vs. placebo) produced significantly higher BDNF in both healthy and depressed participants (medium effect size). Crucially, in patients who received ayahuasca, higher BDNF at 48 h correlated negatively with MADRS (i.e., more BDNF → fewer symptoms); this association was absent on placebo. Remission was best predicted by fewer prior failed antidepressant trials rather than by BDNF or treatment arm [57]. A complementary analysis linked within-session and 48 h biology: among ayahuasca-treated patients, smaller in-session salivary-cortisol increases predicted higher serum BDNF at 48 h, especially in clinical responders, supporting an inverted-U relationship whereby moderate (not excessive) stress activation optimises neurotrophic recovery [74].

The second is inflammation (CRP/IL-6). Before treatment, depressed patients showed elevated CRP versus controls, with no group difference in IL-6; CRP correlated negatively with serum cortisol (consistent with hypocortisolaemia contributing to low-grade inflammation) [68]. At 48 h post-dose, CRP fell substantially after ayahuasca—by ~26.6% in patients and ~32.4% in controls—while IL-6 did not change. Among depressed ayahuasca recipients, the magnitude of CRP reduction correlated positively with MADRS improvement at 48 h (greater CRP drop → greater symptom relief) [68]. In a separate RCT, neither CRP nor IL-6 exhibited moderation by acute subjective intensity, reinforcing that inflammatory change did not depend on hallucinatory strength [74].

The second is the HPA axis (cortisol) and mood coupling. At baseline, TRD patients displayed hypocortisolaemia and a blunted awakening salivary-cortisol response relative to controls (plasma cortisol 15.12 ± 1.73 µg/dL vs. 19.52 ± 1.37 µg/dL; awakening AUC 49.4 ± 8.3 cm^2^ vs. 62.5 ± 6.3 cm^2^). A single ayahuasca dose elicited a marked acute salivary-cortisol rise (~99% in patients; ~147% in controls), whereas the placebo did not. By 48 h, the awakening response normalised in ayahuasca-treated patients (becoming statistically similar to healthy controls who received ayahuasca), while plasma cortisol remained unchanged; these hormonal shifts did not correlate directly with MADRS reductions in that dataset [73]. However, in a mechanistic follow-up, larger within-session mood improvement (greater acute MADRS drop during dosing) predicted higher serum cortisol at 48 h in ayahuasca-treated patients—interpreted as HPA normalisation linked to emotional relief during the session [74].

Notably, the total HRS score (overall “hallucinatory intensity”) did not moderate BDNF, cortisol, CRP, IL-6, or the cortisol awakening response, whereas acute emotional and physiological responses (moment-to-moment mood change; moderate cortisol activation) did [74]. Together, these findings indicate that psilocybin/ayahuasca-associated antidepressant effects are accompanied by neurotrophic upregulation (↑ BDNF), anti-inflammatory shifts (↓ CRP), and HPA axis re-regulation; critically, how patients feel and physiologically respond during dosing (emotional improvement with moderate arousal) better forecast these biological markers than the intensity of perceptual alterations per se [57,68,73,74].

### 2.7. Cognitive and Process-Level Mediators

Across clinical and naturalistic datasets, antidepressant improvement was closely coupled to changes in process variables—principally reductions in experiential avoidance, increases in psychological flexibility and connectedness, and shifts in maladaptive cognition (rumination, thought suppression)—with precise, replicable associations to symptom change. In two prospective cohorts of planned psychedelic use, experiential avoidance (BEAQ) fell markedly from baseline to 2–4 weeks (Study 1: d = 0.9–1.5; Study 2: d = 0.7–0.9), in parallel with large QIDS decreases (Study 1: d ≈ 1.4–2.6; Study 2: d = 1.0–2.1). Crucially, reductions in avoidance correlated with reductions in depression at every time point (ρ = 0.32–0.52) and with diminished suicidal ideation (ρ = 0.15–0.46), with no rebound between weeks 2 and 4 [58]. An ACT-embedded, placebo-controlled crossover in MDD converged: psychological flexibility (AAQ-II) improved more after psilocybin than placebo and tracked symptom change with exceptional precision—QIDS improvement correlated ρ = 0.88 (*p* < 0.001) with AAQ-II gains and ρ = −0.64 (*p* = 0.014) with increased “accept without judgment”; these mind–symptom couplings were absent in the placebo condition [67]. A randomised psilocybin-versus-escitalopram trial demonstrated formal mediation: within the psilocybin arm, decreases in experiential avoidance significantly mediated improvements in clinician-rated and self-reported depression, well-being, and trait anxiety, whereas no such mediation appeared in the SSRI arm; between-group contrasts confirmed stronger indirect effects under psilocybin. Serial models indicated a directional pathway—↓ avoidance → ↑ connectedness → improvement—for well-being and depression/anxiety outcomes. Predictively, ego dissolution and psychological insight during dosing forecast the largest drops in avoidance, while mystical-type and emotional breakthrough scores did not relate to avoidance change [78].

Cognitive style shifts provided an additional mechanistic bridge from acute experience to outcome. In the same head-to-head RCT, psilocybin produced greater 6-week reductions than escitalopram in rumination (time × condition F(1,56) = 4.58, *p* = 0.037; psilocybin Δ = −7.76, *p* < 0.001, d = 0.63) and thought suppression (time × condition F(1,57) = 5.88, *p* = 0.019; Δ = −9.70, *p* < 0.001, d = 0.87). Among responders, both drugs reduced rumination, but only psilocybin responders reduced thought suppression (time × condition × response F(1,54) = 8.42, *p* = 0.005; Δ = −13.95, *p* < 0.001, d = 0.91). Within the psilocybin group, depression improvement correlated with declines in rumination (r = 0.48, *p* = 0.007) and suppression (r = 0.49, *p* = 0.01); mechanistically, greater ego dissolution (r = −0.44 for rumination; r = −0.41 for suppression) and higher psychological insight (r = −0.69; r = −0.56, both *p* < 0.001) predicted these cognitive gains, whereas emotional breakthrough and challenging experience showed weak or null associations [76].

Broader field data reinforce these process links. In a large prospective web study, psychological flexibility increased after psychedelic use (AAQ-II 21.56 → 20.09 at 2 weeks and 20.22 at 4 weeks; F(2,1138) = 3.83, *p* < 0.05) alongside robust QIDS reductions (5.98 → 3.65 → 3.64; F(2,1138) = 69.6, *p* < 0.05). Changes in flexibility correlated with changes in depression at 2 and 4 weeks (r = 0.31; r = 0.21, both *p* < 0.05), and higher emotional breakthrough and mystical scores modestly predicted greater flexibility gains (r = −0.17; r = −0.15, *p* < 0.05). Notably, a retreat setting yielded larger flexibility improvements than non-retreat use (planned contrast *p* = 0.043, 95% CI [−2.75, −0.04]); baseline inflexibility was the strongest predictor of subsequent gain (F(1,271) = 73.92, *p* < 0.001) [81]. Finally, although acute “emotional breakthrough” did not mediate avoidance change in the RCT [78], in a large naturalistic sample it was the only experiential predictor of 2-week depression improvement when modelled alongside mystical and challenging experiences (β = −0.201, *p* = 0.039)—underscoring that emotional processing and insight-driven reductions in avoidance/rigidity are the most consistent cognitive process conduits for psilocybin’s antidepressant effects [64].

### 2.8. Personality Change and Its Relation to Outcomes

Across TRD and MDD samples, psilocybin was associated with adaptive, multi-domain personality shifts that tracked specific features of the acute experience and, in places, downstream symptom change. In TRD given two psilocybin sessions (10 mg, 25 mg), neuroticism decreased and extraversion and openness increased at 3 months (conscientiousness rose at a trend level; agreeableness unchanged). Critically, insightfulness during dosing predicted both the reduction in neuroticism and the increase in extraversion, while spiritual experiences correlated with extraversion gains; patients with higher baseline neuroticism tended to show less improvement in depressive symptoms, and greater baseline openness weakly predicted more intense positive acute experiences (e.g., blissful state) [55]. In a larger randomised head-to-head trial (psilocybin therapy, PT, vs. escitalopram, ET), 6-week personality change favoured broad improvement in both arms but was wider under PT: within the psilocybin group, neuroticism fell (B = −0.63), introversion declined (B = −0.38), disagreeableness (B = −0.47) and impulsivity (B = −0.40) decreased, while openness (B = 0.23), conscientiousness (B = 0.30), and absorption (B = 0.32) increased. In the escitalopram arm, neuroticism (B = −0.38), disagreeableness (B = −0.26), and impulsivity (B = −0.35) decreased, and openness (B = 0.28) and conscientiousness (B = 0.22) increased, but introversion (B = −0.20) and absorption (B = 0.09) showed no significant change. At 6 months, reductions in neuroticism persisted in both groups (psilocybin B = −0.47; escitalopram B = −0.46), with a sustained increase in agreeableness in the psilocybin arm (B = 0.41); between-condition differences were not statistically significant, aside from a trend toward higher absorption with psilocybin (B = 0.23, *p* = 0.037, below the prespecified α for non-hypothesised outcomes). Notably, expectancy selectively shaped escitalopram personality change—higher escitalopram expectancy predicted greater neuroticism decreases (β = −0.01 per unit, *p* = 0.002) and conscientiousness increases (β = 0.01, *p* = 0.004)—whereas psilocybin personality outcomes were not predicted by psilocybin expectancy [72]. Converging naturalistic data from a structured psilocybin programme showed that neuroticism declined from 27.0 → 24.2 immediately after dosing and 23.7 at 3 months, with openness and conscientiousness increasing after treatment (extraversion/agreeableness unchanged). Moderators linked these trait shifts to clinical improvement: the ability to surrender and emotional engagement during dosing predicted gains across depression, anxiety, PTSD, and personality indices; within the MEQ-30, transcendence of time/space/self most strongly moderated improvements in depression/anxiety and in openness/conscientiousness, while post-session personal strength and appreciation for life (PTGI) tracked better mood outcomes and higher openness [82]. Collectively, these findings indicate that psilocybin-related personality changes (reduced neuroticism; increased extraversion, openness, and conscientiousness) are partly determined by acute insight/spiritual facets, appears less contingent on expectancy than SSRI-related trait change, and align with clinical improvement patterns in which insightfulness, surrender, and self-transcendent qualities during dosing forecast both trait adaptation and symptom relief [55,72,82].

### 2.9. Naturalistic and Population-Based Evidence (Predictors, Mediators, and Harms)

In prospective and survey-based cohorts, depressive symptoms fell rapidly and substantially, and specific predictors and mediators consistently accounted for who improved most. In a prospective naturalistic study, mean QIDS decreased by −4.40 at 2 weeks (SE = 0.32, *p* < 0.001; d = 1.18) and −4.17 at 4 weeks (*p* < 0.001; d = 1.13), with the largest absolute and relative reductions in those starting with moderate or severe depression (moderate: 12.6 → ~7.0; severe: ~18.5 → 6.3; effect sizes in the moderate–severe stratum d = 2.4 at 2 weeks and d = 2.1 at 4 weeks). Linear mixed models identified three independent predictors: a medicinal motive (β = 0.316, *p* = 0.002) and higher dose (β = 0.196, *p* = 0.029) predicted greater QIDS improvement, whereas previous psychedelic experience predicted smaller gains (β = 0.213, *p* = 0.033). When acute experience measures were modelled together, only emotional breakthrough predicted 2-week improvement (EBI β = −0.201, *p* = 0.039), while mystical-type (MEQ) and challenging (CEQ) scores were not significant, likely due to collinearity (r ≈ 0.5) with EBI [64].

Findings generalise to large clinician and population samples. Among 228 psychiatric prescribers, PHQ-9 fell 6.03 → 3.02 (*p* < 0.001; d = 0.60), GAD-7 6.11 → 2.74 (*p* < 0.001; d = 0.68), HERO well-being 32.73 → 40.14 (*p* < 0.001; d = 0.84), and suicidality 0.36 → 0.14 (*p* < 0.001; d = 0.32). An exploratory factor (“mystical/connectedness”—awe, nature/universe connection, compassion, gratitude) explained 60.4% of variance (α = 0.94) and independently predicted better outcomes (e.g., depression r = 0.38, anxiety r = 0.36, suicidality r = 0.26, well-being r = −0.57, resilience r = −0.50; all *p* < 0.001). A total of 13.2% reported ≥1 harm, though improvements predominated overall [79]. In a 2510-person community survey, mean PHQ-9 dropped 10.7 → 4.7, GAD-7 9.3 → 3.6, and HERO 28.0 → 39.3 (all *p* < 0.001; d ≈ 1.07–1.10). Benefits scaled with lifetime psychedelic uses in a sigmoidal fashion (steep gains over ~1–10 uses then plateau), with meaningful improvement even after a single use. Substance comparisons showed no PHQ-9/GAD-7 differences between psilocybin and LSD; ayahuasca users reported slightly greater well-being (small effect), and ketamine users smaller gains (likely higher baseline severity). Three stable change dimensions (transformative/mystical, emotional/physical regulation, and pro-social motivation) together accounted for approximately 60% of the variance and each independently predicted reduced depression and anxiety, as well as improved well-being. Harms were reported by 13% (most often increased cannabis, cigarette smoking, or alcohol misuse) and attenuated benefits when present [80].

Process-level mediators paralleled clinical trials. Two longitudinal naturalistic cohorts showed large drops in experiential avoidance (Study 1 d = 0.9–1.5; Study 2 d = 0.7–0.9) alongside sharp QIDS reductions (Study 1 d ≈ 1.4–2.6; Study 2 d = 1.0–2.1), with decreases in avoidance correlating with decreases in depression (ρ = 0.32–0.52) and suicidal ideation (ρ = 0.15–0.46); no rebound occurred between 2 and 4 weeks [58]. In a prospective web study, psychological flexibility (AAQ-II) improved (21.56 → 20.09 → 20.22; F(2,1138) = 3.83, *p* < 0.05) alongside QIDS (5.98 → 3.65 → 3.64; F(2,1138) = 69.6, *p* < 0.05); AAQ-II change correlated with QIDS change at 2 and 4 weeks (r = 0.31; r = 0.21, *p* < 0.05). Greater emotional breakthrough and mystical scores predicted larger flexibility gains (r = −0.17; r = −0.15, *p* < 0.05), and retreat settings yielded bigger flexibility improvements than non-retreat use (planned contrast *p* = 0.043, 95% CI [−2.75, −0.04]); baseline inflexibility was the strongest predictor of gain (F(1,271) = 73.92, *p* < 0.001) [81].

Naturalistic clinical programmes echo these determinants and highlight moderators. In an open-label, therapist-supported psilocybin programme, depression, anxiety, PTSD, and neuroticism decreased and remained improved at 3 months; MEQ-30 averaged ~60% of maximum (“complete mystical-type”), EBI averaged 71/100, surrender 82/100, and personal significance 86/100. Better outcomes were linked to greater surrender and emotional engagement during dosing; within MEQ, transcendence of time/space/self most strongly moderated improvements in depression/anxiety and increases in openness/conscientiousness. History of abuse predicted larger PTSD/neuroticism reductions; ongoing professional support enhanced long-term anxiety reduction; lower alcohol use was associated with bigger gains; younger age predicted larger anxiety decreases [82]. In BIPOC adults reporting psychedelic experiences that alleviated racial-trauma sequelae (n = 313), traumatic stress (d = −0.45), depression (d = −0.52), anxiety (d = −0.53), and stress (d = −0.32) all fell (*p* < 0.001); canonical correlation linked greater mystical and insight (and fewer challenging experiences) to larger symptom reductions (Rc = 0.52, *p* < 0.001). Notably, 73% reported that the index experience occurred ≥1 year prior, suggesting durability [77].

### 2.10. Ayahuasca Trials: Clinical Effects and Mechanistic Signals

Across randomised, placebo-controlled trials, a single ayahuasca session produced rapid antidepressant effects accompanied by coordinated neurotrophic, inflammatory, and HPA axis changes. In a double-blind parallel RCT (n = 29), ayahuasca reduced depression versus placebo on the MADRS at 1–7 days, with between-group effect sizes increasing from d = 0.84 (days 1–2) to d = 1.49 (day 7). HAM-D at day 7 likewise favoured ayahuasca (*p* = 0.019, d = 0.98). Response (≥50% MADRS reduction) was 64% on ayahuasca vs. 27% on placebo; remission (MADRS ≤ 10) showed a trend (36% vs. 7%). Acute dissociative/perceptual changes (CADSS/BPRS) were transient and did not predict improvement. Among responders, stronger HRS perception scores correlated with symptom reduction, and higher MEQ-30 transcendence of time/space correlated inversely with residual depression, suggesting selective experiential mediators within the ayahuasca arm [56].

Neurotrophin data indicate a rapid BDNF increase tied to symptom relief and shaped by cortisol milieu. In a double-blind RCT (patients with TRD and healthy controls), baseline serum BDNF did not track depression severity, but hypocortisolaemia predicted lower BDNF; among eucortisolaemic volunteers, cortisol correlated negatively with BDNF—consistent with an inverted-U cortisol–BDNF relation. At 48 h post-dose, BDNF was significantly higher after ayahuasca than placebo (medium effect). In patients receiving ayahuasca, higher BDNF correlated negatively with MADRS (more BDNF → fewer symptoms), an association absent on placebo. Remission was best predicted by fewer prior failed antidepressants, not by BDNF per se [57].

Inflammatory profiles shifted toward an anti-inflammatory state. Before dosing, patients showed elevated CRP versus controls (IL-6: no group difference) and a negative CRP–cortisol correlation, consistent with hypocortisolaemia-linked low-grade inflammation. At 48 h post-ayahuasca, CRP fell ~26.6% in patients and ~32.4% in controls, whereas IL-6 did not change; among depressed ayahuasca recipients, larger CRP reductions correlated with greater MADRS improvement. Changes in inflammatory markers did not covary with shifts in cortisol or BDNF [68].

HPA axis measures showed acute activation followed by normalisation of circadian dynamics. At baseline, patients exhibited hypocortisolaemia and a blunted awakening salivary-cortisol response (plasma cortisol 15.12 ± 1.73 µg/dL vs. 19.52 ± 1.37 µg/dL in controls; awakening AUC 49.4 ± 8.3 cm^2^ vs. 62.5 ± 6.3 cm^2^). Roughly 1 h 40 min post-ayahuasca, salivary cortisol rose sharply (~99% in patients; ~147% in controls) but not after placebo. By 48 h, the awakening response normalised in ayahuasca-treated patients (plasma cortisol unchanged); these cortisol shifts did not directly correlate with MADRS in that dataset [73].

Linking session dynamics to biology, a mechanistic analysis found that larger within-session mood improvement (greater acute MADRS drop during dosing) predicted higher serum cortisol at 48 h (interpreted as HPA re-regulation) in ayahuasca-treated patients, while smaller in-session salivary-cortisol increases predicted higher 48 h BDNF, particularly among clinical responders, again supporting an inverted-U arousal–neurotrophin relationship. Notably, total HRS “intensity” did not moderate BDNF, cortisol, CRP, IL-6, or the cortisol-awakening response, and CRP/IL-6 showed no session-intensity moderation effects [74].

### 2.11. Global Clinical Efficacy Signals (Context for Moderators)

Across modern trials, psilocybin (and, separately, ayahuasca) produced large, rapid, and often durable antidepressant effects that set the backdrop against which determinants and moderators operate. In a randomised, waiting list-controlled MDD trial, clinician-rated depression fell from a mean GRID-HAMD 22.8 at baseline to 8.0 at week 1 and 8.5 at week 4 in the immediate-treatment arm (between-group Cohen’s d = 2.2 at week 5 and d = 2.6 at week 8; all *p* < 0.001). Self-reported QIDS-SR dropped from 16.7 at baseline to 6.3 the day after session 1 and 6.0 at week 4 (d = 3.1, *p* < 0.001). Clinical response was 67% at week 1 and 71% at week 4; remission was 58% and 54%, respectively. Four-week improvements correlated tightly with session meaning (r = −0.70, *p* < 0.01), psychological insight (r = −0.60, *p* < 0.01), and spiritual significance (r = −0.57, *p* < 0.01), moderately with MEQ30 (r = −0.41, *p* < 0.05), and not with challenging experience (CEQ27) [59]. In TRD (open-label, n = 20), QIDS-SR16 showed very large effects at 1 week (d = 2.2) and 5 weeks (d = 2.3), and sustained benefits at 3 and 6 months (d = 1.5 and 1.4; all *p* < 0.001); at 5 weeks, nine responded and four remitted, with “unity/spiritual/bliss/insightfulness” during dosing predicting larger 5-week improvement (e.g., r = −0.57 for insightfulness) [50]. In severe TRD (single 25 mg; n = 12), MADRS changes were −19.4 (week 1), −15.8 (week 3), and −17.2 (week 12), all large (d ≈ 2–3), with 67% response/42% remission at week 3 and 58% response/25% remission at week 12; early benefit correlated with oceanic boundlessness at week 1 (r = −0.68, *p* = 0.016), and comorbid PTSD attenuated outcomes (PTSD main effect and PTSD × time) [61]. In veterans with severe TRD (baseline MADRS 35.3), a single 25 mg dose yielded a mean −23-point MADRS reduction at week 3 (d = 2.23, *p* < 0.001), 60% response/53% remission at week 3, and 47%/40% at week 12; outcomes were not detectably influenced by age, sex, race, or PTSD, and were not mediated by 5D-ASC scores [51].

Outside primary MDD/TRD, two double-blind crossover trials in life-threatening cancer showed large, durable reductions in depression and anxiety after high-dose psilocybin: at 6 months, 78% met depression response (GRID-HAMD) and 65% remission; for anxiety, 83% responded and 57% remitted. MEQ30 scores strongly predicted and mediated therapeutic gains across mood and well-being endpoints, independent of drug intensity ratings [62,65]. A long-term MDD study (two sessions, 20 and 30 mg/70 kg) found GRID-HAMD falling from 22.8 to ~8 at all follow-ups through 12 months, with 75% response and 58% remission at one year (Cohen’s d = 2.0–2.6 across time points); spiritual meaning/insight robustly tracked well-being, with limited, time-specific links to depression change [52].

### 2.12. fMRI Results

Across nine studies, psilocybin treatment yielded rapid and sustained clinical improvements that were accompanied by convergent, multi-level changes in brain perfusion, task-evoked responsivity, large-scale network organisation, and dynamical properties of functional activity in fMRI results. In treatment-resistant depression (TRD), perfusion imaging showed post-treatment decreases in cerebral blood flow (CBF), most prominently in temporal cortex and the left amygdala; the magnitude of amygdala CBF reduction correlated with greater symptom improvement, linking limbic down-shift to clinical benefit [84]. Resting-state analyses in the same cohort revealed increased default mode network (DMN) integrity—particularly strengthened ventromedial prefrontal (vmPFC) coupling with bilateral inferior lateral parietal cortex—which did not track immediate symptom change but predicted sustained response at five weeks. In contrast, decreased parahippocampal–prefrontal connectivity predicted better longer-term outcomes, and no reliable changes were detected in amygdala resting connectivity [84]. Mediation analyses suggested that the acuteness of the psychedelic experience (including “peak/mystical” qualities) related to the extent of parahippocampal decoupling, providing an experiential bridge between dosing and subsequent network reconfiguration [84].

Task fMRI during emotional face viewing consistently indicated enhanced amygdala responsivity after therapy alongside a loosening of prefrontal–amygdala coupling. An open-label TRD study found increased right amygdala activation to fearful and happy faces, with the fear > neutral increase predicting reductions in BDI and QIDS scores up to three weeks; responders and remitters showed increased amygdala reactivity, whereas non-responders showed decreases [85]. In partially overlapping participants, psychophysiological interaction analyses showed decreased vmPFC–right amygdala connectivity during face processing (especially for fearful and neutral expressions). This reduction was not directly tied to depression severity but predicted lower rumination at one week, and it co-occurred with increased coupling of both amygdala and vmPFC to occipito-parietal visual areas that tracked improvements in depression and anxiety and was strongest among clinical responders/remitters [92]. Together, these task results indicate a shift toward greater limbic responsivity to affective cues with reduced prefrontal constraint, alongside enhanced engagement of visual cortices with emotion networks.

At the level of large-scale network topology, resting-state analyses across an open-label TRD trial and a double-blind randomised comparison with escitalopram converged on a characteristic post-psilocybin “desegregation” signature. Whole-brain modularity decreased one day after dosing—i.e., networks became more globally integrated—and this change predicted symptom improvements up to six months in the open-label sample [86]. Psilocybin decreased within-DMN connectivity while increasing its integration with executive and salience systems, suggesting more open communication among higher-order networks typically over-segregated in depression; escitalopram produced milder clinical effects without significant network-level changes [86]. In the randomised cohort, improvements in mood correlated with increases in dynamic network flexibility, especially within the executive network, indicating an enhanced capacity of regions to reassign their functional allegiances over time [86]. Complementing these findings, a generative modelling study of hierarchical organisation reported opposite reconfigurations for psilocybin vs. escitalopram: psilocybin decreased global directedness (hierarchical “flattening”), while escitalopram increased it; these effects were dissociable from remission rates (64% psilocybin vs. 30% escitalopram) and were supported by machine learning separation of pre-/post-treatment brain states with ~0.89 accuracy [91]. Within treatments, responders exhibited distinct regional trophic shifts: after psilocybin, widespread cortical and subcortical territories—especially cingulate, hippocampus, and amygdala—migrated upward in the hierarchy, whereas escitalopram improvements were linked to hierarchical normalisation within anterior cingulate and striatal reward regions [91]. Baseline hierarchical features predicted escitalopram response with 85% accuracy, underscoring the prognostic utility of pre-treatment brain organisation [91].

Predictive modelling using pre-treatment functional connectivity reinforced that the initial network architecture carries information about subsequent clinical courses. Across two datasets, early improvement (≤5 weeks) after psilocybin was best predicted by connectivity patterns anchored in visual systems—primary and extrastriate—and their links to frontal and temporal cortices (accuracies near 0.9), with additional contributions from DMN and executive § connections; in contrast, longer-term change (24 weeks) was preferentially predicted by salience network connectivity within frontal, insular, and limbic nodes. These patterns partially generalised across datasets and were replicated when samples were combined [87].

Analyses of time-resolved dynamics provided orthogonal evidence that psilocybin shifts the brain toward a more flexible, metastable regime that distinguishes responders from non-responders. Probabilistic metastable substates (PMSs) derived from fMRI revealed no pre-treatment group differences, but post-treatment responders showed a selective reconfiguration of one substate, an effect not captured by static connectivity [88]. Personalised whole-brain oscillator models tuned to empirical pre-treatment data indicated that mildly increasing regional oscillatory drive most effectively pushed the system from a “depressed” to a “healthy” post-psilocybin configuration observed in responders, whereas adding noise or driving toward a depressive pattern degraded the fit [88]. Regions with the greatest modelled “transition potential” in responders—temporal pole, rolandic operculum, fusiform, supplementary motor area, inferior/angular parietal, supramarginal, inferior frontal opercular/orbital, and parahippocampal gyri—overlapped with territories rich in 5-HT_2_A and 5-HT_1_A receptors; correlations between regional transition potential and receptor density were significant (ρ = 0.23, *p* = 0.032 for 5-HT_2_A; ρ = 0.28, *p* = 0.007 for 5-HT_1_A), and absent for other serotonergic targets or the transporter [88].

Finally, studies leveraging music as an emotionally salient probe showed convergent limbic–DMN rebalancing and selective enhancement of sensory-affective responsivity. Subjectively, participants reported greater music-evoked pleasure and peacefulness with reduced sadness after therapy, improvements that inversely tracked anhedonia and persisted for up to three months [89]. Neurally, music listening shifted from pre-treatment coupling to post-treatment decoupling between nucleus accumbens (NAcs) and DMN nodes, consistent with reduced top-down control of reward systems; although NAc–DMN changes did not directly correlate with pleasure ratings, the behavioural gains in hedonia were robust [89]. In parallel, the amplitude of low-frequency fluctuations (ALFF) increased post-treatment in bilateral superior temporal gyrus and supramarginal gyrus during music, while resting ALFF decreased in medial frontal cortex; the magnitude of music-evoked ALFF increases correlated with multiple 5D-ASC dimensions—including ego dissolution, visionary restructuralisation, auditory alterations, and vigilance reduction—and with the global intensity of the psychedelic state after correction, tying the quality of the acute experience to later sensory–emotional neural responsivity [90].

In aggregate, the neuroimaging fMRI results depict a coherent pattern: psilocybin moves depressed brains toward less segregated, more dynamically flexible and hierarchically flattened configurations; relaxes prefrontal constraint over limbic and reward systems while enhancing bottom-up engagement with affective and sensory input; and yields predictive signatures at baseline (visual/DMN/executive and salience networks; hierarchical features) that stratify early vs. sustained responses. These effects are supported across perfusion, task-evoked activation/connectivity, resting-state topology, dynamical modelling, and receptor-anchored analyses, and they align with dose session phenomenology as an intermediate variable linking acute psychedelic quality to durable neural and clinical change [84,85,86,87,88,89,90,91,92].

### 2.13. EEG Results

In the study [93], the EEG analyses focused on auditory-evoked theta power (4–8 Hz), a frequency band associated with long-term potentiation (LTP) and cortical plasticity. Participants underwent an auditory tetanic stimulation paradigm designed to elicit LTP-like changes in neural activity. The key outcome was the difference in theta power before and after this stimulation, both 24 h and two weeks after placebo and psilocybin administration. The main result was a significant increase in overall theta power two weeks after psilocybin, compared with both the placebo condition and earlier time points. The magnitude of theta power roughly doubled relative to the baseline. The most striking finding was the negative correlation between changes in theta power and depression severity. Specifically, increases in theta power from 24 h to two weeks post-psilocybin were significantly correlated with decreases in GRID-HAM-D-17 scores (Spearman’s ρ = −0.57, *p* < 0.03). Participants showing the largest increases in theta power experienced the greatest clinical improvement.

## 3. Discussion

**Figure 3:**
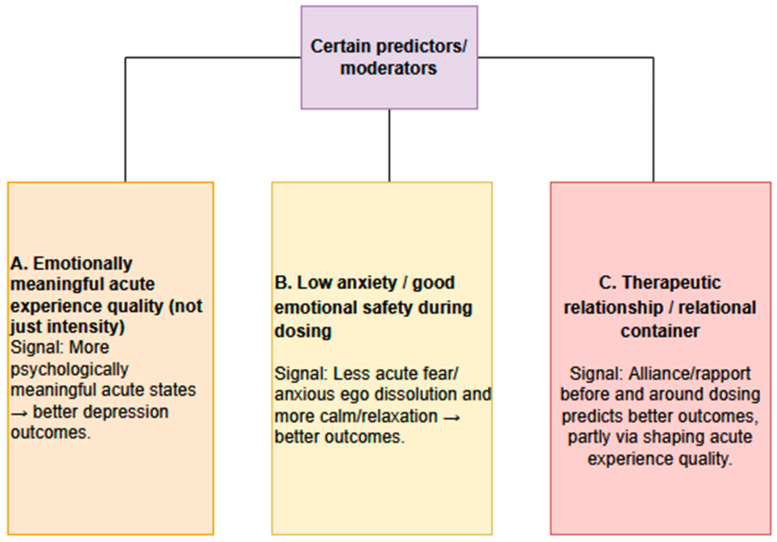
Certain predictors.

**Figure 4:**
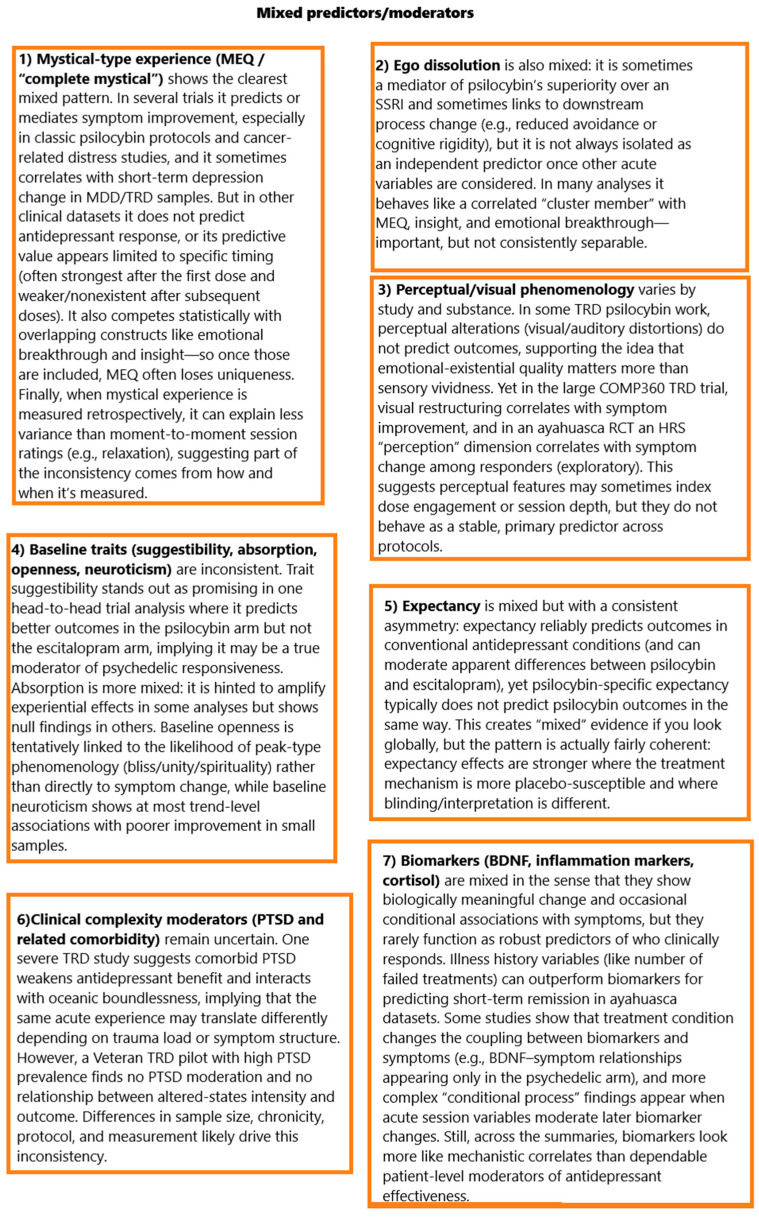
Mixed predictors.

**Figure 5:**
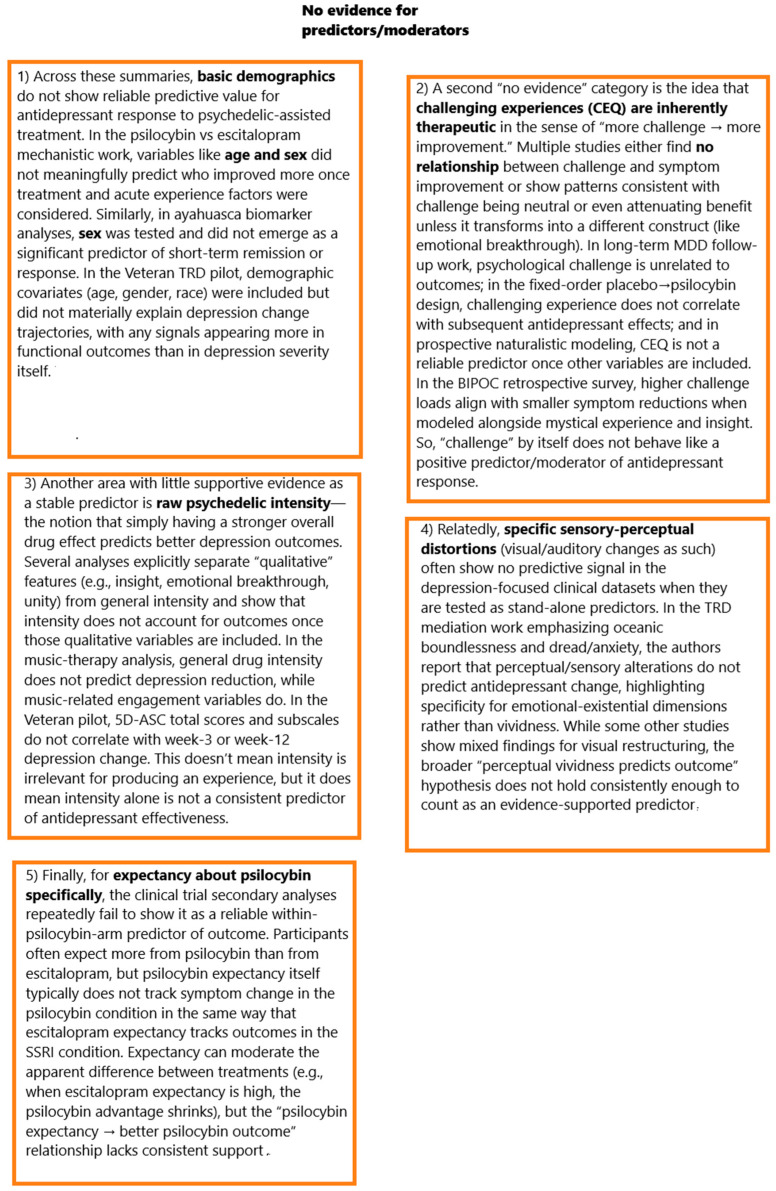
No evidence for predictors.

Research on the effectiveness of psychedelics in treating depression is probably the most widely explored area of research concerning this group of substances. Numerous reviews and meta-analyses have demonstrated moderate to high efficacy in treating depression. However, to date, no work has been published examining the predictors and moderators of treatment effectiveness. Filling this research gap is crucial, as it allows for answers to important questions such as differences in treatment response and how to design studies to maximise the effectiveness of psychedelics in treating depression. Below, we discuss the findings gathered in this review within the broader context of psychedelic research. The positive predictors are shown in Figure 3, the mixed predictors in Figure 4, and the null predictors in Figure 5.

Across studies, a pattern—that the quality of the acute psychedelic state (unitive/mystical qualities, ego dissolution, and especially emotional breakthrough) predicts clinical benefit while anxious, dysphoric states attenuate it—aligns with a converging mechanistic account that bridges receptor pharmacology, systems-level brain dynamics, and emotion learning theory.

Classic psychedelics act primarily via 5-HT2A receptor activation; in humans, psilocybin’s 5-HT2A occupancy scales with subjective intensity, tying neuropharmacology to conscious experience. Blocking 5-HT2A with ketanserin largely abolishes LSD’s subjective and connectivity effects, underscoring that what is experienced depends on this receptor gate [94]. Yet “intensity” alone is insufficient: experiential content (e.g., oceanic boundlessness, insight) tracks outcomes far better than sensory fireworks. On systems scales, psychedelics transiently relax high-level priors and increase brain network flexibility (REBUS/anarchic brain) [95], with ego dissolution linked to expanded global connectivity and DMN decoupling—precisely the kinds of state shifts that could permit perspective change [96]. Together, these data support a finding that unitive/insightful states (rather than generic intensity) are therapeutically germane.

EB indexes a specific process—approach to previously avoided affect and the emergence of workable meaning—that maps closely onto established change mechanisms [97]. In exposure science, durable symptom reduction hinges on inhibitory learning and expectancy violation rather than mere habituation [98]; in memory-reconsolidation models, activating a maladaptive emotional memory and juxtaposing disconfirming experience enables updating/erasure [99]. Psychedelic-occasioned EB is a potent way to create those conditions (high prediction error, safety, affective openness). Validations of the Emotional Breakthrough Inventory (EBI) emphasise EB as a distinct mediator, and complementary lab work shows that psychedelic states reduce amygdala reactivity to social threat while increasing positive affect—exactly the affective terrain in which learning updates occur [100,101].

An observation that emotional engagement with music predicts benefit fits the robust literature: psychedelics reliably amplify music-evoked emotion and imagery and modulate parahippocampal/limbic connectivity during listening. Recent neuroimaging further shows that heightened music-evoked responses after therapy relate to reduced anhedonia [102,103]. Practically, this suggests that therapist-guided, personally meaningful music can scaffold EB and insight when patients are most receptive.

At the cellular level, psychedelics promote rapid neuritogenesis and spine formation in cortex [19] and may directly sensitise BDNF/TrkB signalling [20], yielding a transient plasticity window. In vivo two-photon work confirms persistent increases in dendritic spine density after a single psilocybin dose [104]. These neuroplastic shifts likely amplify the lasting impact of what is learned during the acute session—hence the outsized predictive power of EB/insight compared with generic intensity.

High DED (default ego dissolution, an anxiety-laden, dysregulated state) consistently weakens outcomes in the data. Ego dissolution is often considered a core phenomenological feature of psychedelic states and has been linked to therapeutic outcomes in some datasets. Survey work on challenging experiences shows mixed or even adverse associations, with severity predicting risk and not reliably portending a growth-absent supportive context [105]. The large literature converges on specific PTSD-related alterations in threat learning and regulation—hyperreactive amygdalae, hypoactive ventromedial prefrontal cortex (vmPFC), impaired hippocampal/context processing, and difficulties with fear inhibition/extinction [106]. These circuit features help explain why some patients with comorbid PTSD obtain less benefit from a single high-dose psychedelic session: the session can acutely amplify arousal while the underlying capacity to update fear memories and down-regulate threat remains constrained, making “letting go” and emotional processing harder in the dosing window. Mechanistically, psychedelics might counteract these constraints when conditions align, because 5-HT_2_A agonism can enhance plasticity in circuits central to fear updating. In rodents, psilocybin and other serotonergic psychedelics facilitate fear extinction and memory updating [21,107,108]; in healthy humans, psilocybin reduces amygdala reactivity to threat cues [101], consistent with a tilt toward safety learning. Thus, heterogeneity in PTSD (e.g., dissociative vs. fear-dominant subtypes) likely gates whether the same drug-assisted session produces corrective learning or defensive disengagement [109].

PTSD is also neuroendocrinologically diverse: subgroups show that HPA axis dysregulation (often relative hypocortisolism), which can impair the consolidation of corrective emotional experiences during therapy and might reduce carry-over of session gains to everyday life [110]. This could further moderate psychedelic outcomes in some cohorts.

Outside depression, mystical/unitive qualities and insight predict outcomes in substance use and cancer-related distress. In smoking cessation, “mystical-type” experience correlates with long-term abstinence [111,112]; in alcohol use disorder, psilocybin reduces heavy-drinking days and exploratory analyses link mystical-type components to better drinking outcomes [113]. Cancer distress work included in this review similarly finds that MEQ-assessed mystical experience mediates improvements in anxiety, mood, and meaning, independent of raw drug intensity. This triangulation strengthens the claim that experiential quality is the therapeutic signal. Mystical/unitive qualities are not a vague metaphor but a measurable construct that captures internal/external unity, noetic quality/meaning, sacredness, and ineffability, typically quantified using instruments such as the MEQ-30 (with factors including “mystical/unitive” content, positive mood, transcendence of time/space, and ineffability) [114]. In the depression trials summarised here, higher oceanic boundlessness/”unity” and related facets (e.g., spiritual significance, personal meaning, insightfulness) repeatedly tracked larger symptom reductions, and in some datasets mystical-type measures statistically mediated therapeutic outcomes even after accounting for overall drug intensity, supporting the view that experiential quality can be the proximal therapeutic signal rather than the non-specific “strength” of intoxication. Mechanistically, a pars on is that unitive experiences reflect an acute state of reduced defensive self-focus and increased connectedness/meaning making, which can enable cognitive-affective reappraisal and value reconsolidation during a window of heightened plasticity; in this framing, mystical/unitive qualities may function as a phenomenological marker of successful engagement with the therapeutic process (safety, openness, emotional approach) rather than a necessary ingredient in every case. This also fits mixed findings in naturalistic and “real-time” ratings where relaxation or other moment-to-moment indices sometimes outperform retrospective MEQ totals, implying that (i) ceiling effects, (ii) collinearity with emotional breakthrough/insight, and (iii) measurement timing can obscure unique mystical contributions when multiple acute constructs are modelled together.

The finding that MEQ predicted benefit only after the first dose (despite more “complete” mystical experiences later) likely reflects ceiling effects and shrinking between-person variance: once most participants achieve a high MEQ, between-person differences carry less information. It also hints that early EB/insight may “set the trajectory,” after which integration and therapy—not ever-higher mystical scores—drive continued gains. Moment-to-moment indices (e.g., relaxation, emotional approach) may therefore outperform retrospective totals in later sessions. This is consistent with evidence that within-session affective engagement, rather than generic intensity, best predicts change.

Many measures (MEQ, EBI, CEQ) are retrospective and confounded with one another; high-frequency, in-session sampling (e.g., micro-phenomenology or continuous affect ratings) plus physiological markers could sharpen mediation tests. Mechanistically, distinguishing the contributions of receptor-level plasticity from top-down belief relaxation (REBUS) will require designs that orthogonally manipulate the affective content, suggestibility, and 5-HT2A signalling (e.g., ketanserin challenges with therapist-guided EB). Finally, given PTSD’s apparent moderation, trials that explicitly target inhibitory learning/reconsolidation principles under psychedelic conditions may clarify how to deliver EB safely in trauma-laden populations.

Across datasets, alliance and music function less like background “supports” and more like levers that shape the very contents and functions of the psychedelic state that, in turn, drive symptom change. This is precisely what decades of “set and setting” scholarship would predict: extra-pharmacological factors help determine how loosened priors (and heightened malleability) are canalised into either insight and relief or anxiety and stalemate. In other words, the context does not merely add to drug effects—it selects and formats them [115,116].

A credible, task-focused bond is an antidote to threat and a scaffold for exploration. Social neuroscience shows that supportive presence down-regulates neural threat responding (e.g., spouse handholding during threat reduces activity in key salience/aversive systems), consistent with Social Baseline Theory’s proposal that humans outsource regulation to trusted others. Polyvagal theory similarly frames safety cues (prosody, eye contact, attunement) as signals that permit ventral vagal engagement and emotional openness. In session, these ingredients reduce defensive arousal and open the window for the high-entropy psychedelic state to reorganise experience rather than amplify fear [117,118,119].

The broader psychotherapy literature corroborates the causal direction implied in our path models. Early alliance reliably predicts later symptom improvement in individual patient-data and session-by-session analyses; importantly, a substantial portion of this relation sits at the therapist level, i.e., some clinicians reliably form stronger alliances and achieve better outcomes (therapist effects) [120,121,122]. That maps neatly onto current findings that pre-dosing task/engagement predicts more transformative acute states.

Two further process studies illuminate how a strong alliance potentiates “emotional breakthrough.” First, empathy meta-analyses show a robust association with outcome, implying that the kind of attuned responding you observe post-session is not an epiphenomenon but an active ingredient. Second, studies of behavioural/physiological synchrony (voice, movement, electrodermal activity) link moment-to-moment attunement to stronger alliance and better outcomes—precisely the micro-mechanism by which guides may help patients “stay with” difficult affect long enough to transform it [123,124,125].

A pattern—expectancy strongly shaping SSRI outcomes but not psilocybin, while suggestibility (not absorption) moderates psilocybin benefit—is exactly what we would predict when we place both treatments inside contemporary models of (i) placebo/nocebo neurobiology, (ii) predictive-processing accounts of drug action, and (iii) the psychology and neuroscience of suggestion.

Across medicine, expectations can amplify or erase pharmacology: in open–hidden experiments, positive expectancy doubles remifentanil analgesia and negative expectancy abolishes it, with matching changes in pain circuits; in Parkinson’s disease, mere expectation triggers striatal dopamine release and symptomatic benefit. These paradigms show that slow-acting or subtly felt drugs are unusually permeable to belief effects. SSRIs fit that profile: their early, largely preconscious shifts in emotional processing require weeks of downstream learning and social feedback to translate into mood change, leaving a wide window for expectancies (and nocebo-driven side effects/adherence) to steer outcomes. This accords with the robust expectancy–response links seen for SSRIs in the current data [126,127,128,129,130,131,132,133,134].

Macrodose psilocybin produces striking, salient state changes that swamp coarse, pre-session beliefs. Two features matter. First, the acute psychedelic state is dominated by bottom-up affective/imaginal input under relaxed high-level priors (REBUS), reducing the precision-weighting of abstract, propositional expectations (e.g., “I think I’ll improve 60%”). Second, blinding in psychedelic RCTs is notoriously fragile; yet, within the psilocybin arm, explicit expectancy typically shows weak associations with outcome, implying that what drives benefit is not pre-trial belief per se but how contextual cues shape the quality of the acute state (mystical/insightful vs. anxious/dysphoric). Reviews of masking integrity in psychedelic trials, together with theoretical accounts, recommend measuring expectancy but caution against over-attributing outcomes to it when the pharmacology so powerfully determines the state content [95,135,136,137].

Unlike generic expectancy, suggestibility indexes openness to, and enactment of, suggestions and context—precisely the psychological lever therapists and music can pull during dosing. Classic studies and modern replications show that LSD increases suggestibility in healthy volunteers (without simply boosting cued imagery), implying that richer, sustained suggestions (therapeutic framing, music-guided emotional approach) can exert an outsized influence on phenomenology and, downstream, clinical change. Convergent hypnosis research links suggestibility to a reproducible fronto-cingulate control motif (DLPFC↔dACC coupling), supporting a trait capacity to reconfigure percept, affect, and agency in response to suggestion—i.e., the very machinery psilocybin renders highly plastic. This maps cleanly onto current findings that a higher baseline suggestibility potentiated psilocybin (but not SSRI) outcomes [138,139,140,141].

Trait absorption reliably predicts the vividness and type of acute psychedelic effects—especially mystical/unitive facets—in healthy volunteer and naturalistic cohorts, yet it is a blunter predictor of therapeutic change. That asymmetry reconciles our null absorption–outcome associations with prior psychopharmacology: absorption helps people immerse, but immersion alone does not guarantee the direction of learning (breakthrough vs. overwhelm) or its consolidation into life change. In other words, absorption may load on state intensity, while suggestibility gates state shaping—which is what matters for clinical gain. Current trait-by-mediator effects (absorption and suggestibility amplifying mystical/ego dissolution mediations) fit this division of labour [142,143,144].

Lower baseline use of positive emotion words predicted larger downstream gains fits decades of computational linguistics showing that depressed speech/text contains fewer positive and more negative emotion words and greater first-person singular—digital markers of low positive affect and self-focus. Patients who start “colder” affectively may simply have more headroom for increases in positive emotion and social approach after a session that successfully evokes safety and connection [145,146]. Personality signals align with this. High baseline neuroticism generally tracks more persistent negative affect and stress reactivity; conversely, psychedelic-occasioned states can acutely increase psychological openness and pro-sociality, with downstream structural plasticity (e.g., dendritic spine growth) offering a cellular substrate for durable shifts. These mechanisms plausibly mediate observed reductions in neuroticism and increases in extraversion/openness when dosing elicits insight/acceptance rather than anxiety.

Motivational “set” matters. Across health domains, autonomous (self-endorsed) motivation predicts better engagement and outcomes; in psychedelic care, clear self-healing intentions likely translate into stronger task-focused behaviour before/during/after dosing (preparation, in-session effort, and integration work), amplifying benefits [147,148].

Why might prior psychedelic experience predict smaller gains? Two complementary mechanisms are plausible. First, rapid pharmacodynamic tolerance and cross-tolerance at 5-HT2A (receptor desensitisation/down-regulation) can blunt subjective/affective response if exposures are recent or frequent. Second, learning theories emphasise that novelty and prediction error drive synaptic change and memory updating; a first profound psychedelic session delivers a large “surprise” signal, while subsequent sessions may produce less mismatch—and therefore less plastic change—unless set/setting are redesigned to re-introduce novelty and expectancy violation [149,150,151].

**Figure 6:**
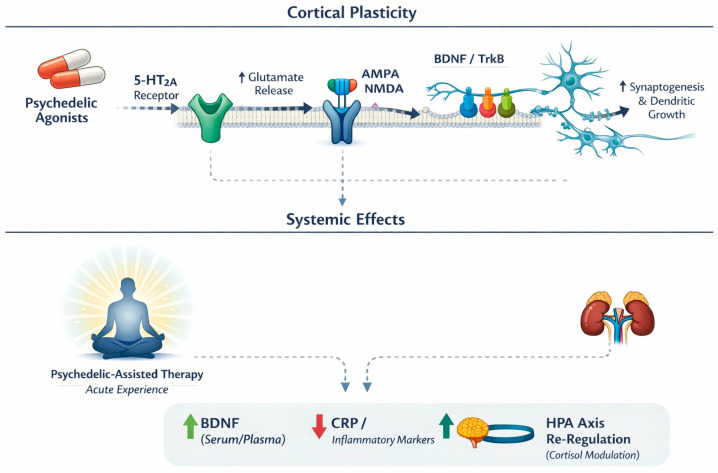
Biological effects of psychedelics in depression. ↓ decrease, ↑ increase.

Ayahuasca findings—↑ BDNF, ↓ CRP, and HPA axis re-regulation with little movement in IL-6—fit a coherent, multi-level mechanism linking 5-HT2A/tryptamine pharmacology to plasticity, stress physiology, and immune tone. These effects are shown in Figure 6.

Psychedelics reliably induce rapid, lasting synaptic remodelling: in vivo two-photon imaging shows that psilocybin increases dendritic spine density and size within 24 h (persisting ≥1 month), while preclinical work demonstrates “psychoplastogenic” neurite/spine growth that depends on TrkB and mTOR signalling. More recently, some psychedelics (e.g., LSD/psilocin) were shown to directly bind TrkB and potentiate BDNF signalling, offering a receptor-level bridge from 5-HT2A engagement to trophic cascades [152]. In ayahuasca specifically, β-carbolines such as harmine add complementary biology by inhibiting DYRK1A and stimulating the proliferation of human neural progenitor cells—changes that would synergise with the BDNF upregulation observed at 48 h [153]. Together, these data make the BDNF rise a plausible mediator (and/or permissive marker) of antidepressant learning after dosing.

The inverted-U pattern—smaller in-session cortisol surges and better mood shifts forecasting higher BDNF—aligns with classic stress learning biology. Across human and animal studies, glucocorticoid effects on memory and plasticity are dose- and timing-dependent: moderate, phasic elevations facilitate consolidation/extinction and retrieval shaping, whereas too little (or too much) impairs updating [154,155,156]. This helps explain why ayahuasca’s acute salivary-cortisol spike and subsequent normalisation of the awakening response at ~48 h can be adaptive when emotional relief occurs in-session—i.e., when stress energy is channelled into corrective learning rather than dysregulation.

Multiple lines of evidence support direct immunomodulation by classic tryptamines. Selective 5-HT2 agonism (e.g., (R)-DOI) suppresses TNF-α/NF-κB-driven inflammatory cascades in vitro and in vivo [157,158,159]; tryptamines in ayahuasca (DMT/5-MeO-DMT) activate the sigma-1 receptor to shift human dendritic cells [160] and cerebral organoids toward anti-inflammatory transcriptional states [161]. Consistent with this, clinical studies show CRP reductions after ayahuasca (and exploratory CRP/IL-6 decreases after psilocybin in healthy volunteers) [25]; current findings that CRP dropped while IL-6 was unchanged likely reflect biomarker kinetics and sampling windows: IL-6 is highly diurnal with a short half-life (t1/2 ≈ 20–30 min) [162,163], whereas CRP (hepatic, t1/2 ≈ 19 h) integrates upstream cytokine signals over time and is therefore more stable at 24–72 h assays [164]. The negative cortisol↔CRP coupling noted is also physiologically coherent, as relative hypocortisolism can permit low-grade inflammation [165].

Across the datasets, the most reliable conduits from the acute state to clinical change were process shifts—especially less experiential avoidance, greater psychological flexibility and connectedness, and reductions in rumination and thought suppression. These are not idiosyncratic to psychedelics; they map onto well-established, transdiagnostic mechanisms in clinical science and contemporary cognitive neuroscience.

The psychological flexibility framework (spanning acceptance, defusion, present-moment awareness, values, and committed action) predicts distress across diagnoses and improves with intervention; meta-analytic and mechanistic work repeatedly tie gains on flexibility to symptom relief. Our tight BEAQ/AAQ–symptom couplings sit squarely in this literature [166,167,168].

Viewed through learning theory, decreases in avoidance enable the very updates patients need. The inhibitory learning account of exposure shows that durable change depends less on within-session fear dampening and more on building new “safety” predictions under conditions of moderate arousal—precisely the pattern current ACT-embedded crossover and session-level analyses revealed [7,169]. Psychedelic sessions that cultivate approach and acceptance likely maximise inhibitory learning and reconsolidation-style memory updating during a window of heightened plasticity.

Rumination is a transdiagnostic vulnerability that amplifies and prolongs negative affect; reducing it is a robust pathway to relief. Thought suppression is even trickier: decades of work document its paradoxical rebound, with greater intrusions and worse mood after attempts to push thoughts away. Our finding that psilocybin—but not SSRI—responders uniquely decreased suppression dovetails with this literature and helps explain the stronger downstream effects when avoidance gives way to acceptance-based processing [170,171,172].

Neurally, these cognitive styles are anchored in self-generated thought systems: the default mode network (DMN) supports internally oriented, self-referential mentation, and its dynamics track ruminative processing. By loosening entrenched top-down priors (REBUS) and remodelling large-scale connectivity—for example, LSD-induced global connectivity increases that scale with ego dissolution and psilocybin-evoked desynchronisation lasting beyond the drug state—psychedelics may transiently free cognition from rigid DMN-dominated loops, enabling reappraisal and acceptance to take hold [95,96,173,174].

Serial models from this review (↓ avoidance → ↑ connectedness → ↓ symptoms) align with the broader evidence that belonging/connectedness is a powerful determinant of mental and physical health, and that self-transcendent states (e.g., awe) reliably expand perceived connection and pro-sociality. Gains in felt connection—toward others and nature—are repeatedly linked to well-being, offering a plausible bridge from in-session openness to durable mood change [175,176,177,178].

Psychedelic modulation of depression-related neurocircuitry is most parsimoniously interpreted within a 5-HT2A-centric framework that links molecular engagement to systems-level reconfiguration and, ultimately, to psychotherapy-relevant psychological processes. Classic psychedelics are high-affinity 5-HT2A receptor agonists; occupancy of cortical 5-HT2A sites by psilocybin’s active metabolite tracks subjective intensity, and 5-HT2A density is highest across the heteromodal cortex that anchors transmodal association networks. These pharmacological and anatomical facts scaffold contemporary theories—Entropic Brain and REBUS—which propose that psychedelics relax the precision of high-level priors, increasing receptivity to bottom-up information and enabling belief updating during therapy [16,95,179,180].

At the macro-scale, the post-treatment decreases in modularity and increases in network flexibility fit a broader pattern seen across acute psychedelic states: a shift from segregated to more globally integrated dynamics. Under LSD and psilocybin, global connectivity increases and canonical boundaries between systems loosen—changes that correlate with ego dissolution—and whole-brain models and receptor-informed control theory suggest that stimulating cortex rich in 5-HT2A receptors flattens the brain’s energy landscape, facilitating transitions among network states. The present finding that lower modularity after dosing predicts durable symptom relief is therefore consistent with a mechanistic chain in which 5-HT2A-driven desegregation enables the system to reconfigure into more flexible, less crystallised modes [96,181,182,183].

**Figure 7:**
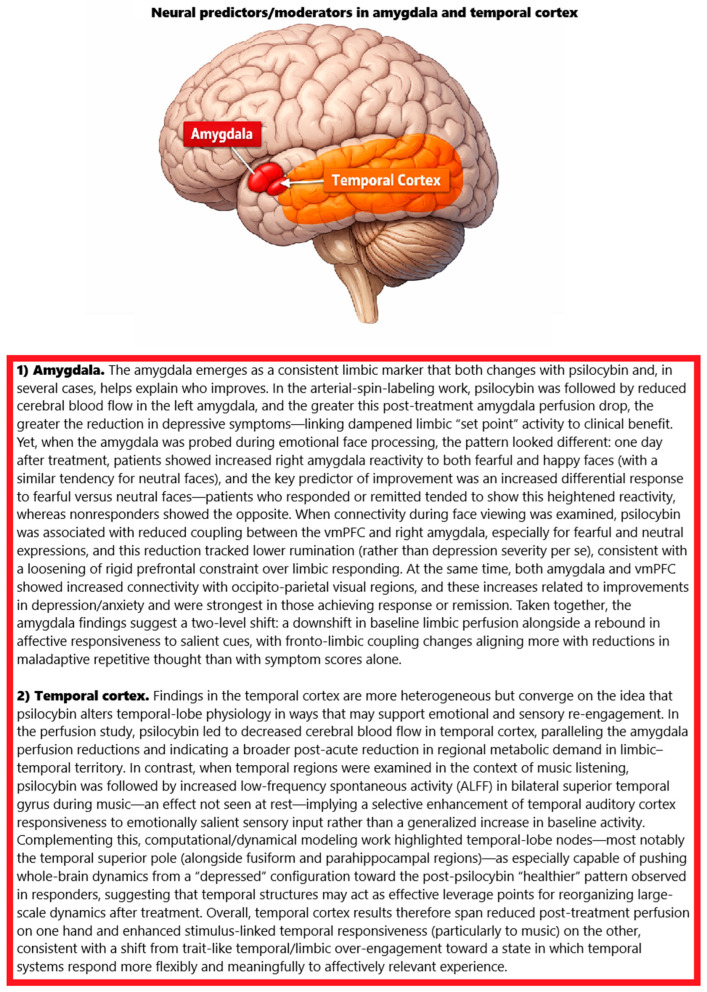
Neural predictors in amygdala and temporal cortex.

A complementary affective mechanism is suggested by the convergence of increased amygdala responsivity with decreased vmPFC → amygdala coupling during emotional face viewing. In depression, conventional serotonergic antidepressants tend to blunt amygdala reactivity to negative affective cues (acutely in volunteers and over weeks in patients), whereas psilocybin appears to transiently “lift the brake,” loosening top-down constraint while increasing bottom-up salience processing. Within a predictive-processing account, that loosened constraint is precisely what REBUS predicts, temporarily relaxing high-level precision to permit the revision of maladaptive priors (e.g., self-critical schemas) when emotionally salient material is engaged during therapy. Thalamocortical findings under LSD further support a gating account in which 5-HT2A stimulation retunes cortico-striato-thalamo-cortical loops to admit more sensory-affective information into cortical processing streams [94,95,184,185,186]. Neural predictors of effectiveness of psychedelics in the amygdala and temporal cortex are presented in Figure 7.

Findings with music as an affective probe dovetail with this view of “bottom-up reopening.” Music reliably engages mesolimbic reward circuitry—including caudate during anticipatory phases and nucleus accumbens (NAcs) during peak pleasure—and depression is marked by weakened corticostriatal reward coupling and anhedonia. Post-psilocybin increases in music-evoked pleasure alongside reduced NAc–DMN coupling are thus interpretable as a release from self-referential, inhibitory control over reward systems, allowing hedonic signals to register and update affective priors. That pattern coheres with evidence that reduced NAc connectivity characterises MDD and that inflammation-linked disruptions of corticostriatal coupling track anhedonia; psilocybin’s decoupling of NAc from DMN during music may represent a functional “unclenching” of reward processing [187,188,189,190].

Baseline predictors centred on visual and fronto-temporal connections make mechanistic sense given the receptor topography and acute psychedelic phenomenology. The visual cortex is especially perturbed by LSD/psilocybin, 5-HT2A density is high in associative cortex (including occipital hubs), and acute studies show strengthened coupling between visual areas and higher networks during altered imagery. Individuals whose visual–frontal/temporal scaffolding is already better integrated may therefore be more “perturbable,” translating the drug’s receptor-level action into large-scale network recalibration and early clinical gains [16,191].

The longer-horizon prominence of salience network predictors fits with theories assigning the insula–cingulate system a role in adjudicating competition between internal (DMN) and external (executive/attention) modes. Depression is frequently characterised by DMN hyper-integration and maladaptive coupling with subgenual cingulate that supports rumination. By first desegregating networks and then re-establishing more adaptive control by salience hubs, psilocybin may help shift the operating point from rigid self-referential capture toward contextually appropriate switching—consistent with dynamic flexibility increases seen here and elsewhere. Notably, reports across the depression literature show altered DMN coupling (often increased within-DMN and with subgenual cingulate), but directionality can vary by subtype and stage; therapeutic “re-integration” after a psychedelic perturbation may involve selective restoration of long-range coherence (e.g., vmPFC–parietal) against a backdrop of reduced maladaptive cingulo-limbic coupling [192,193,194].

Timescale helps reconcile apparent tensions (e.g., acute desegregation vs. certain post-treatment increases in within-DMN integrity). Acute dosing produces widespread desynchronisation and integration across networks; after the acute window, the perturbed system appears to resettle into configurations that are less segregated overall yet capable of coherent long-range coordination when needed. This “elastic” recalibration is seen in longitudinal precision-mapping after psilocybin (massive acute FC disruption with progressive post-acute normalisation) and in models showing that 5-HT2A-weighted stimulation lowers the control energy required to move between network states. In short, the drug session provides a high-entropy perturbation; psychotherapy and integration capitalise on the ensuing plastic window to bias the settling point toward healthier attractors [174,182].

Finally, dynamical and receptor-anchored evidence ties these network effects to plausible microcircuit mechanisms. 5-HT2A receptors are enriched on apical dendrites of layer-V pyramidal neurons, where their activation boosts glutamatergic drive and modulates local excitation–inhibition balance; connectome-harmonic work shows frequency-selective reweighting of cortical modes under LSD, and biophysical models map pharmacologically induced reorganisation onto the brain’s neurotransmitter landscape. The present overlap between regions with high post-treatment “transition potential” and cortical 5-HT2A (and 5-HT1A) maps is therefore expected if psilocybin’s primary action is to increase the brain’s capacity to explore and stabilise alternative dynamical regimes [17,195,196,197].

A useful way to read the theta finding is as a delayed systems-level signature of synaptic repair and re-coupling across cortico-hippocampal circuits after 5-HT2A-biassed stimulation. Acutely, psychedelics that agonise 5-HT2A receptors increase cortical excitability and disrupt canonical large-scale network coupling; this “decompression” of network dynamics tracks well with human EEG/MEG and pharmacology showing 5-HT2A-dependent alterations in population rhythms and connectivity during the psychedelic state. Over days, however, the same receptor engagement drives plasticity cascades (BDNF/TrkB–mTOR, AMPAR trafficking, spinogenesis) that outlast the acute experience and change how those networks talk to each other. In animals and ex vivo tissue, psilocybin rapidly increases dendritic spine formation and presynaptic markers; in vivo autoradiography in pigs demonstrates higher synaptic density one week after a single dose (SV2A upregulation) together with down-regulation of cortical 5-HT2A binding, a pattern consistent with a plasticity “afterglow.” These subacute synaptic gains offer a mechanistic substrate on which large-scale oscillations can reorganise [198,199,200].

Theta (4–8 Hz) is a natural carrier for that reorganisation because it coordinates long-range information flow between the hippocampus, medial prefrontal cortex, and striatum—the same networks recruited for memory updating, cognitive control, and emotion regulation. Human intracranial/EEG-fMRI studies consistently show that successful recollection and top-down control ride on increases in hippocampal–prefrontal coupling in the theta/low-alpha range; when theta is phase-aligned across these nodes, communication becomes more efficient and plasticity is facilitated. Recent causal work using closed-loop, phase-locked stimulation in neurosurgical patients further shows that enhancing neocortical inputs precisely at hippocampal theta phase boosts cortico-hippocampal communication, underscoring theta’s mechanistic role rather than it being a mere epiphenomenon [201,202,203].

Major depression, by contrast, is marked by lower synaptic density and disconnected networks. Human PET with [^11^C]UCB-J links the severity of depressive symptoms to reduced SV2A (a presynaptic density marker), which co-occurs with aberrant functional connectivity. From this vantage, the post-psilocybin rise in theta is parsimoniously read as an emergent property of restored “wiring”: as synapses are added and strengthened in hubs like the hippocampus and medial PFC, those regions can once again phase-lock at theta, enabling more adaptive prediction error handling, affective updating, and cognitive flexibility—the very processes that clinical interviewing captures as relief from rumination and negativity bias [204].

The Skosnik et al. trial ties these strands together in patients: two weeks after psilocybin (but not placebo), auditory-evoked theta power approximately doubled and individual increases in theta tracked the magnitude of symptom relief. The absence of classic tetanus-induced LTP in their MDD sample actually sharpens the interpretation: rather than showing stimulus-locked LTP in a lab assay, patients exhibited a global gain in theta-mediated excitability/coupling at rest and during simple sensory processing—exactly what one would expect if plasticity had been implemented “offline” over days and then expressed as better network synchronisation. In other words, theta is the readable systems-level echo of molecular and structural changes that unfolded after dosing.

## 4. Materials and Methods

### 4.1. Study Design

This study was conducted as a scoping review. Scoping reviews are particularly appropriate for mapping the extent, range, and nature of evidence on a given topic, especially when concepts are complex or heterogeneous—as is the case with predictors of treatment effectiveness in psychedelic therapy for depression.

### 4.2. Research Question

This review aimed to answer the following question: What patient- or treatment-related factors have been proposed or evaluated as predictors of treatment effectiveness in psychedelic-assisted therapy for depression, and what is the current state of evidence regarding their validity?

### 4.3. Eligibility Criteria

Studies were eligible for inclusion if they met the following criteria:

Population: Adults diagnosed with depressive disorders, including major depressive disorder (MDD), persistent depressive disorder, or treatment-resistant depression.

Intervention: Psychedelic-assisted therapy using substances such as psilocybin, LSD, ayahuasca, DMT, or mescaline.

Comparator: Studies were searched for that did or did not compare psychedelics with another intervention or placebo.

Outcome: The study examined any predictor (e.g., demographic, clinical, neurobiological, psychometric, or psychological) associated with treatment response or effectiveness.

Study Design: Randomised controlled trials, prospective or retrospective observational studies, secondary analyses of trial data, or exploratory predictive modelling studies.

Language: English.

Publication Type: Peer-reviewed journal articles only. Conference abstracts, editorials, reviews, and grey literature were excluded.

### 4.4. Information Sources and Search Strategy

A systematic search was conducted in the following electronic databases: PubMed (MEDLINE), Embase, PsycINFO, and Web of Science. The initial search was conducted in September 2025, with an update performed in October 2025. The search strategy combined controlled vocabulary (e.g., MeSH terms) with free-text keywords relating to the following:

Psychedelics (e.g., “psilocybin,” “LSD,” “ayahuasca”)

Depression (e.g., “depressive disorder,” “major depression,” “treatment-resistant depression”)

Predictors (e.g., “predict*,” “moderator,” “biomarker,” “individual differences”)

An example search string used for PubMed was as follows:

(“psilocybin” OR “LSD” OR “ayahuasca” OR “DMT” OR “mescaline”) AND (“depression” OR “major depressive disorder” OR “treatment-resistant depression”) AND (“predict*” OR “moderator” OR “biomarker” OR “prognostic factor”).

The reference lists of all included articles and relevant reviews were also hand-searched to identify additional eligible studies.

### 4.5. Study Selection

All identified records were imported into a reference management software and de-duplicated in EndNote 21 (Clarivate). Two internal reviewers independently screened titles and abstracts for potential relevance. Full texts of potentially eligible studies were then retrieved and assessed against the inclusion criteria. Any disagreements were resolved through discussion or consultation with a third reviewer.

## 5. Conclusions

This scoping review indicates that the process-level features of the acute psychedelic session—and the context shaping them—best predict the antidepressant response. Across study designs, emotional breakthrough, mystical/unitive qualities, and ego dissolution-enabled perspective shifts are associated with greater and more sustained symptom relief, while anxious or dysphoric states reduce the therapeutic benefits. In short, experiential quality—not sheer intensity—carries the therapeutic signal.

Elements of set and setting are active levers. A stronger therapeutic alliance and intentionally curated, resonant music enhance the likelihood of transformative experiences and subsequent clinical improvement. Expectancy predicts SSRI outcomes but not psilocybin’s, whereas suggestibility moderates the psilocybin benefit; absorption relates more to phenomenological vividness than clinical change.

Baseline moderators show smaller, mixed effects. PTSD comorbidity may attenuate gains in some TRD cohorts; extensive prior psychedelic exposure can mean smaller incremental benefit. Medicinal motive and higher dose help mainly insofar as they foster the curative experiences described earlier. Exploratory signals (e.g., pre-treatment speech valence, higher neuroticism) warrant prospective testing; demographics add little.

Multi-level biology converges with this picture. Psychedelics shift brain networks toward less segregation and greater flexibility, loosen rigid prefrontal constraint, and show EEG and peripheral biomarker changes consistent with enhanced plasticity, providing a plausible bridge from session quality → neural change → clinical outcome.

Overall, who benefits most depends less on who the patients are and more on what they experience and how skilfully the context channels that experience. Engineering the set, setting, and support to reliably elicit emotional breakthrough and insight emerges as the most evidence-aligned pathway for achieving durable antidepressant change.

## Figures and Tables

**Table 1:** Biological, behavioural and neuroimaging moderators/predictors of antidepressant effectiveness in psychedelic depression studies.

Study	Design/Sample	Psychedelic	Depression Outcome Window	Moderators/Predictors (Effectiveness)
[46]	TRD, n = 20, psilocybin-assisted therapy	Psilocybin 10 mg + 25 mg	QIDS-SR baseline → 1 d/1 w/5 w	Higher OBN (mystical/unitive) + lower DED (anxiety/cognitive impairment) strongly predicted larger symptom reductions; perceptual/sensory changes did not predict outcomes; “complete mystical” (OBN > 0.6) linked to higher response across measures.
[47]	TRD + bipolar II, n = 31, 1–3 sessions	Psilocybin 25 mg	MADRS change 2 w after each dose	Mystical experience predicted improvement only after dose 1 (significant after controlling baseline severity); not significant after later doses (limited power).
[48]	RCT: psilocybin therapy vs. escitalopram	Psilocybin 25 mg ×2 vs. quasi-placebo + SSRI	Depression factor to week 6	Mystical experience & ego dissolution mediated psilocybin’s superior effect (beyond intensity/visuals/music impact). Within psilocybin: higher mystical experience, emotional breakthrough, emotional engagement with music → larger improvement. Trait absorption & suggestibility strengthened mediation (moderation). Demographics (age/sex) not predictive.
[49]	Phase II dose-finding RCT, TRD, n = 233	COMP360 25/10/1 mg (single dose)	MADRS to 3 weeks	Strongest predictors of improvement: oceanic boundlessness, visual visual restructuralisation, EBI (esp. at 25 mg). Anxious ego dissolution/reduced vigilance, weakly related.
[50]	TRD, open-label, n = 20	Psilocybin 10 mg + 25 mg	QIDS to 1 w/5 w/3–6 m	Acute experience quality predicted longer-term benefit: unity, spiritual experience, blissful state, insightfulness (USB composite) correlated with larger improvement.
[51]	Swiss medical-use PAT: patients vs. healthy controls (n = 28 + 28)	LSD or psilocybin (clinical programme)	MADRS pre/post (immediate, ~1 w)	Relaxation during the session was the strongest predictor of antidepressant response. Mystical-type experience did not predict; real-time ratings explained more variance than retrospective MEQ. Dose/drug type not related.
[52]	RCT (waitlist control), MDD n = 27 (24 completers), 12-month follow	Psilocybin 20 & 30 mg/70 kg	GRID-HAMD to 12 months	Session ratings of meaning/spiritual significance/insight predicted well-being more consistently than depression; only at ~4 w did meaning/spiritual significance correlate with depression improvement; challenge not related.
[53]	Placebo then psilocybin fixed-order (within-subject), TRD-MDD	Psilocybin 0.3 mg/kg (max 35 mg)	GRID-HAMD/QIDS early weeks	MEQ and challenging experience during psilocybin were not correlated with antidepressant change; placebo session MEQ correlated with improvement (interpreted as expectancy/context).
[54]	TRD, open-label, n = 17; ML prediction	Psilocybin 10 mg + 25 mg	QIDS baseline → 5 w	Baseline speech sentiment features predicted responder status (~85% accuracy): responders showed fewer emotional words, especially fewer positive words (AVG positivity most sensitive).
[55]	TRD, open-label feasibility, n = 20	Psilocybin 10 mg + 25 mg	QIDS to 3 months + personality	Acute insightfulness predicted larger ↓ neuroticism and ↑ extraversion; baseline openness tentatively linked to stronger bliss/unity/spiritual aspects; baseline neuroticism trend: higher → less symptom improvement.
[56]	RCT placebo-controlled TRD-MDD (Brazil), n = 29	Ayahuasca single dose	MADRS to day 7	Exploratory predictors: among responders HRS “perception” correlated with greater improvement; MEQ “transcendence of time/space” correlated with greater improvement; other acute symptom ratings not correlated.
[57]	Biomarker analysis within ayahuasca RCT	Ayahuasca vs. placebo	Remission at 48 h (MADRS ≤ 10)	Best predictor of remission: fewer prior failed antidepressant treatments. BDNF did not predict binary remission; ayahuasca condition showed BDNF–symptom coupling (higher BDNF linked to lower MADRS only in ayahuasca group). Baseline cortisol status predicted baseline BDNF (not remission).
[58]	2 naturalistic cohorts (planned psychedelic use), n = 104 & n = 254	Various serotonergic psychedelics	Depression & suicidality to 2–4 w	Key predictor of improvement: reductions in experiential avoidance correlated with reductions in depression and suicidal ideation. Not a formal moderator model; pattern held in mild depression subsample.
[59]	Waitlist-controlled RCT, MDD n = 27 (24 completers)	Psilocybin 20 & 30 mg/70 kg	GRID-HAMD to 4 w	Acute mystical-type, personal meaning, psychological insight associated with greater depression reduction (reported in supplement as predictors/correlates).
[60]	Process model (psilocybin vs. escitalopram trial data)	Psilocybin 25 mg ×2	QIDS to ~6 w	Alliance → rapport → stronger acute experience (EBI/MEQ) → lower depression. Emotional breakthrough often stronger link than MEQ. Mid-treatment severity linked to weaker alliance/rapport before session 2 → worse outcomes (conditioning factor).
[61]	Severe TRD open-label, n = 12	COMP360 25 mg (single dose)	MADRS to week 12	Predictor: higher oceanic boundlessness correlated with better early outcomes (week 1; not robust later). Moderator: comorbid PTSD associated with weaker benefit, esp. later follow-ups; PTSD × OB interaction reported (OB–outcome relation differed by PTSD status).
[62]	Cancer distress crossover RCT, n = 51	Psilocybin high vs. very low	GRID-HAMD/HAM-A to 5 w & 6 m	MEQ mediated dose effects on depression/anxiety outcomes; MEQ associations persisted controlling for intensity. Focused more on experiential mediation than baseline “who benefits” moderators.
[63]	Cancer distress crossover RCT, n = 29	Psilocybin 0.3 mg/kg vs. niacin	Depression/anxiety to weeks	Higher MEQ linked to greater improvement; MEQ mediated treatment effects. Baseline moderators tested (gender, prior hallucinogen use, spirituality, cancer stage) not significant.
[64]	Prospective naturalistic survey, baseline mild depression+, n = 302	Various psychedelics	QIDS to 2–4 w	Larger improvement associated with stronger medicinal motive, higher dose, fewer prior psychedelic experiences. Acute experience model: EBI predicted improvement; MEQ not significant once overlap with EBI considered; CEQ not reliable.
[65]	Secondary analysis of psilocybin vs. escitalopram trial, n = 55	Psilocybin 25 mg ×2 vs. SSRI	Multiple outcomes to 6 w	In psilocybin arm: baseline trait suggestibility robustly predicted greater improvement across outcomes; absorption not predictive. Expectancy predicted outcomes mainly in escitalopram arm, not psilocybin.
[66]	TRD open-label, n = 19; qualitative + rated music variables	Psilocybin 10 mg + 25 mg	QIDS to 1 w post-25 mg	Music experience quality (liking, resonance, openness) predicted larger depression reductions; drug intensity did not. Music variables linked to mystical/insightfulness components.
[67]	Placebo then psilocybin fixed-order, ACT-based	Psilocybin 0.3 mg/kg	QIDS to 16 w	Process predictors: larger depression reduction associated with ↑ psychological flexibility (↓ AAQ-II) and ↑ accept-without-judgement after psilocybin; associations not significant in placebo phase.
[68]	Inflammation biomarkers in ayahuasca RCT	Ayahuasca vs. placebo	MADRS at 48 h	Baseline inflammation or change scores did not predict response/remission well in regression models, even though some within-condition symptom–biomarker relations appeared (e.g., CRP change within ayahuasca group).
[69]	Secondary analysis of [46] trial, n = 24	Psilocybin 20 & 30 mg/70 kg	GRID-HAMD to 12 m	Strong predictor: therapeutic alliance (esp. task subscale) predicted lower depression at multiple timepoints. Alliance also predicted higher peak MEQ and insight, and insight strongly predicted long-term outcomes.
[70]	Prediction using day-after integration session (COMP001 subset)	COMP360 25/10/1 mg	Response at week 3; sustained to week 12	Early predictors: EBI + NLP-derived valence/arousal in patient/therapist speech during integration + dose group predicted response (high accuracy/AUC). More positive & activated language + higher EBI → better outcomes.
[71]	Expectancy moderation letter (trial re-analysis)	Psilocybin vs. escitalopram	HDRS-17 at week 6	Escitalopram expectancy moderated comparative effects: when escitalopram expectancy low → psilocybin advantage larger; when high → difference shrank. Psilocybin expectancy did not moderate.
[72]	Personality change analysis in psilocybin vs. escitalopram trial, n = 59	Psilocybin 25 mg ×2 vs. SSRI	Personality to week 6 & month 6	Moderator pattern: psilocybin expectancy did not moderate change in psilocybin arm; escitalopram expectancy did moderate neuroticism ↓ and conscientiousness ↑ in SSRI arm. Tested acute experience moderators (MEQ/EBI/insight/intensity) for personality change.
[73]	Cortisol regulation in ayahuasca RCT	Ayahuasca vs. placebo	MADRS to 48 h + cortisol indices	Treatment assignment predicted endocrine shifts; baseline cortisol differences by group. Cortisol measures did not predict symptom improvement reliably over short window.
[74]	Conditional process models in ayahuasca RCT	Ayahuasca vs. placebo	MADRS to 48 h + biomarkers	Acute moderators of biomarker relations (not strongly of symptoms): early symptom reduction during session and cortisol response moderated links to D2 biomarkers (e.g., BDNF), while HRS subjective intensity did not moderate biomarker outcomes.
[75]	Survey of psilocybin users: expectancies, not clinical outcomes	Psilocybin (past use)	Expected antidepressant benefit	Predictors of expectancy: emotional breakthrough & ego dissolution explained unique variance; mystical experience not unique once overlap accounted. (Not a clinical effectiveness predictor.)
[76]	Mechanism analysis: rumination/suppression in psilocybin vs. SSRI trial	Psilocybin vs. escitalopram	QIDS to 6 w + cognitive styles	Reduction in rumination tracked response in both arms (general marker). Thought suppression decreased mainly in psilocybin responders (response × condition × time). Within psilocybin: ego dissolution & psychological insight associated with larger decreases in rumination/suppression.
[77]	Cross-sectional retrospective BIPOC survey (non-clinical setting)	Psilocybin/LSD/MDMA	DASS-Depression change (30 d before vs. after)	Larger improvements linked to higher mystical-type + higher insight + lower challenging effects (canonical correlation).
[78]	Mediation/process in psilocybin vs. SSRI trial	Psilocybin vs. escitalopram	Depression, well-being, SI, anxiety at 6 w	Psilocybin effects statistically explained by ↓ experiential avoidance (stronger mediation than SSRI). Within psilocybin: ego dissolution & insight predicted reductions in experiential avoidance; mystical experience & emotional breakthrough did not uniquely predict avoidance change.
[79]	PAWS: prescriber subgroup, retrospective	Various psychedelics	PHQ-9 “before vs. after”	Largest improvements predicted by transformative/mystical/interpersonal change factor (PCQ-derived); fewer negative consequences (NCI-8) → larger gains; age (older) associated with greater improvement; lifetime use frequency not reliable.
[80]	PAWS full sample, retrospective, n = 2510	Various psychedelics	PHQ-9 “before vs. after”	Predictors: higher PCQ transformative factors → greater improvement; negative consequences → smaller gains; lifetime use modestly related with diminishing returns; little consistent effect by preferred agent.
[81]	Prospective naturalistic survey, n = 260	Various psychedelics	QIDS to 2–4 w	Predictors of flexibility gains (linked to depression improvement): higher baseline inflexibility → larger AAQ-II improvement; retreat setting → greater AAQ-II reductions; higher EBI and MEQ → more AAQ-II improvement; demographics/prior use not strong predictors once baseline controlled.
[82]	Naturalistic psilocybin programme, open-label, n = 83	Psilocybin ~25 mg equiv (truffles)	PHQ-9 to 1 w & 3 m	Moderators of larger depression reductions: ability to surrender, more emotionally powerful session, and higher MEQ facets (esp. transcendence/mysticality). Post-session growth facets (personal strength, appreciation of life) predicted more durable improvement.
[83]	Veteran severe TRD open-label pilot, n = 15	COMP360 25 mg	MADRS to 3 w & 12 w	5D-ASC intensity/subscales did not predict depression change; PTSD diagnosis did not significantly moderate trajectory (small sample). Medication restart during follow-up functioned as a marker of reduced durability (classified as non-response thereafter).
[84]	Open-label; TRD; n = 19; 2 sessions with psychological support; fMRI pre + 1 day after 2nd dose	Psilocybin 10 mg + 25 mg (1 week apart)	Symptom change at 1 week; response assessed at 5 weeks (≥50% reduction; 47% responders)	Greater ↓ left amygdala CBF correlated with larger symptom decreases; ↑ DMN RSFC (vmPFC–bilateral ilPC) predicted sustained response at 5 weeks; ↓ PH–PFC connectivity predicted positive long-term outcome; peak/mystical experience intensity mediated ↓ parahippocampal connectivity (linking acute experience → later neural/clinical effects).
[85]	Open-label; moderate–severe TRD; n = 20; emotional faces fMRI task pre + 1 day after 2nd dose	Psilocybin 10 mg + 25 mg (1 week apart)	Depression outcomes at 1 week and up to 3 weeks (BDI, QIDS)	↑ right amygdala reactivity to emotional faces post-treatment; ↑ fearful vs. neutral right amygdala response predicted improvement (BDI/QIDS) at 1–3 weeks; responders/remitters showed increased reactivity vs. non-responders decreased.
[86]	Two trials: (1) open-label TRD; (2) DB-RCT psilocybin vs. escitalopram; resting-state fMRI 1 day after treatment	Psilocybin (vs. escitalopram comparator)	Long-term symptom improvement predicted up to 6 months (open-label); acute effects evident rapidly	↓ Brain network modularity (more global integration) 1 day after predicted symptom improvements up to 6 months; ↓ within-DMN connectivity + ↑ integration with EN/SN; in psilocybin group, mood improvement correlated with ↑ dynamic network flexibility (esp. executive network).
[87]	Predictive modelling (ML) using baseline resting-state fMRI from two trials (open-label TRD + psilocybin vs. escitalopram in MDD)	Psilocybin (prediction of response)	Early improvement up to 5 weeks; long-term improvement at 24 weeks	Baseline FC predictors: visual network connectivity best for early improvement (accuracy ~0.9; stronger visual–frontal/occipital–temporal in responders); baseline DMN + executive control (fronto-parietal) also predicted early response; salience network FC predicted 24-week improvement (frontal/insular/limbic).
[88]	Open-label TRD; responders vs. non-responders; dynamic fMRI analyses + computational modelling; 2-dose protocol	Psilocybin 10 mg + 25 mg (1 week apart)	Response defined at 5 weeks (>50% reduction)	Post-treatment change in a specific probabilistic metastable substate (PMS “substate 3”) observed in responders; dynamic sensitivity simulations identified regions with higher “transition potential” in responders (e.g., temporal pole, operculum, fusiform, SMA, inferior/angular parietal, supramarginal, inferior frontal, parahippocampal); transition potential correlated with 5-HT_2_A and 5-HT_1_A receptor density.
[89]	Open-label TRD; n = 19; fMRI pre + 1 day after; music vs. silence scans; self-ratings (pleasure; GEMS)	Psilocybin 10 mg + 25 mg (1 week apart)	Hedonic/anhedonia effects evident 1 day, sustained up to 3 months	Music-evoked pleasure ↑ and sadness ↓; greater pleasure correlated with ↓ anhedonia (Snaith–Hamilton); ↓ NAc–DMN FC during music post-treatment (mechanistic signal), but FC change not significantly correlated with pleasure (predictor evidence limited).
[90]	Open-label TRD; n = 19; fMRI (rest + music) 1 week before and 1 day after 2nd dose; ALFF analysis	Psilocybin 10 mg + 25 mg (1 week apart)	Depression window not emphasised; neural outcomes pre/post; links to subjective experience (acute)	↑ ALFF during music in bilateral superior temporal gyrus (auditory cortex) post-treatment; ↓ ALFF at rest in medial frontal lobe; intensity/quality of psychedelic state (5D-ASC) correlated with magnitude of ALFF increases (DED, VRS, AUA, VIR, and global ASC index).
[91]	DB-RCT (NCT03429075); n = 42; psilocybin arm vs. escitalopram arm; resting-state fMRI pre + 3 weeks after 2nd dose	Psilocybin 25 mg ×2 (3 weeks apart; with placebo) vs. “microdose” psilocybin 1 mg ×2 + daily escitalopram 10–20 mg	Primary imaging window 3 weeks post-2nd dose; remission rates reported (psilocybin 64% vs. escitalopram 30%)	Treatment-specific brain changes: psilocybin ↓ global directedness (hierarchy “flattening”) vs. escitalopram ↑ directedness; within psilocybin, responders showed regions (cingulate, hippocampus, amygdala) moving “up” hierarchy; baseline hierarchy measures predicted escitalopram responders (ML ~85% accuracy).
[92]	Open-label TRD; n = 19; emotional face viewing connectivity fMRI pre + morning after high dose	Psilocybin 10 mg + 25 mg (1 week apart)	Depression outcomes at 1 week (BDI response/remission), rumination at 1 week and 3 months	↓ vmPFC–right amygdala FC during face processing associated with ↓ rumination at 1 week (not directly depression severity); ↑ amygdala/vmPFC connectivity with occipital–parietal visual areas correlated with improved depression/anxiety and stronger in responders/remitters.
[93]	MDD; n = 19; double-blind, placebo-controlled, within-subject, fixed-order (placebo first, then psilocybin); EEG auditory “tetanus” plasticity marker assessed 24 h and 2 weeks post-dose	Psilocybin 0.3 mg/kg (max 35 mg) (after placebo session); supportive therapy	Depression severity (GRID-HAM-D-17) tracked across 24 h → 2 weeks post-dose (psilocybin vs. placebo)	Key predictor signal: larger increase in evoked theta power (24 h → 2 w after psilocybin) associated with larger GRID-HAM-D-17 reductions over same interval; no dedicated baseline moderator battery reported (demographics/clinical history not emphasised as predictors).

↓ decrease, ↑ increase.

## Data Availability

No new data were created or analysed in this study. Data sharing is not applicable to this article.
